# Design of a novel epitope-based tetravalent subunit vaccine against dengue virus: An immunoinformatic approach

**DOI:** 10.1371/journal.pone.0354891

**Published:** 2026-07-28

**Authors:** Tanbin Jahan Ferdousy, Md Mehedy Hasan Miraz, Tawsif Al Arian, Md Afif Ullah, Md Tahsinul Haque Risat, Most. Afrin Akter, Shouhardyo Kundu, Bidduth Kumar Sarkar, Arghya Prosun Sarkar, Abul Bashar Ripon Khalipha, Sukalyan Kumar Kundu

**Affiliations:** 1 School of Pharmacy, BRAC University, Dhaka, Bangladesh; 2 Department of Pharmacy, Jahangirnagar University, Savar, Dhaka, Bangladesh; 3 Department of Microbiology, University of Rajshahi, Rajshahi, Bangladesh; 4 Department of Microbiology, Jahangirganagar University, Savar, Dhaka, Bangladesh; 5 Department of Pharmacy, Islamic University, Kushtia, Bangladesh; 6 Department of Pharmacy, Bangabandhu Sheikh Mujibur Rahman Science and Technology University, Gopalganj, Bangladesh; Instituto Nacional de Salud Pública: Instituto Nacional de Salud Publica, MEXICO

## Abstract

Dengue imposes a profound global impact, with millions affected annually. Its transmission by *Aedes* mosquitoes poses significant challenges to combat, aggravated by urbanization and climate change. Despite efforts, no impeccable antiviral treatment exists to date, highlighting the urgency for a vaccine. Developing one encounters hurdles like the four distinctive serotypes of the virus and complex immune responses. In this study, we employed an immunoinformatics approach to design an epitope-based tetravalent subunit vaccine aimed at confronting all DENV serotypes. The study contemplates epitope prediction and screening, physicochemical assessment, molecular docking, molecular dynamics (MD) simulations, immune simulations, and *in silico* cloning. The vaccine construct comprises human β-defensin 3 adjuvant and 23 epitopes joined with linkers. Notably, the vaccine has an antigenicity of 0.9319 with 97.35% population coverage worldwide. Molecular docking with toll-like receptor 2 (TLR2) and TLR4 showed promising interactions with lowest energies of −1240.5 kJ/mol (76 members) and −1393.3 kJ/mol (40 members), respectively, exhibiting significant electrostatic, van dar Waals, and other interactions. Molecular dynamics (MD) simulations of 200 nanoseconds revealed the vaccine to be stable and flexible with the receptors. The solvent accessibility and radius of gyration were also in suitable ranges. The three-dose vaccine regimen elicited a robust and durable multi-lineage immune response, characterized by stable B and T-cell memory, sustained surveillance, and high-titer antibody production (IgM/IgG) alongside a pro-inflammatory cytokine profile (IFN-γ and IL-2) for effective antigen clearance. These findings suggest that our vaccine outperforms any other Dengue vaccine developed to date. However, since this study was conducted through *in silico* methods, *in vitro* and *in vivo* validations are required to confirm the vaccine as a potential candidate for clinical trials.

## Introduction

Dengue has posed a significant global health concern in recent decades. Around 4 billion individuals (nearly half of the global population) reside in regions vulnerable to dengue exposure [1]. Every year, roughly 400 million people incur dengue, and around 100 million are affected, whereas about 40,000 people die from severe manifestations [1]. The World Health Organization (WHO) documented more than 5 million dengue cases with over 5,000 deaths in more than 80 territories and five WHO regions in 2023 [2]. Dengue is prevalent in more than 100 countries spanning the American, Southeast Asian, Western Pacific, and African regions. The geographical escalation of dengue is evoked by climate change, urbanization, and increased global travel [3]. WHO has endorsed the use of integrated vector management, along with early and precise diagnosis, to lessen the impact of the disease. Despite these efforts, dengue continues to challenge public health systems worldwide, and no definitive therapeutic or preventive solution has been established.

Dengue virus (DENV) is an RNA virus and a member of the *Flaviviridae* family. The virus is transmitted by mosquitoes of several *Aedes species*, mainly by *Aedes aegypti* [4]. There are four serotypes of the virus—DENV-1, DENV-2, DENV-3, and DENV-4—which are capable of producing independent diseases and may even exhibit immunological cross-reactivity with one another. An individual may experience all four serotypes of DENV in the life span. The viral genome encodes structural proteins (capsid protein, C; precursor membrane protein, prM; and envelope protein, E), and non-structural proteins (NS1, NS2a, NS2b, NS3, NS4a, NS4b, and NS5). The capsid (C) protein plays a crucial role in the packaging of the viral genome. It also interacts with the viral RNA during assembly and release [5]. The precursor membrane (prM) protein functions as a chaperone for the envelope (E) protein, thus ensuring proper folding of the envelope protein and thereby enhancing the immunogenicity [6]. The protein E is essential for attachment and entry of the virus into host cells. It also induces neutralizing and protective antibodies, and thus elicits the immune response of hosts [7].

Among the non-structural proteins, NS1 has various functions such as viral replication, evading the immune response by interfering with the host complement system, and affecting endothelial permeability to exacerbate disease severity [8]. NS2a has an important function in viral RNA replication, assembly of virions, and adjustment of the antiviral reaction of the host [9]. NS2b acts as a co-factor for the NS3 protease, playing a role in viral replication and potentially changing membrane permeability [10]. NS3 acts as a flexible protein necessary for copying the viral genome, performing tasks like RNA helicase and RNA triphosphatase activities, and being involved in viral polyprotein processing with its serine protease function [11]. It also impacts the immune responses of the host and aids in the development of viral pathogenesis. NS4a plays a role in creating the viral replication complex and altering membranes in host cells, which are important for RNA replication and avoiding the host’s antiviral defenses during DENV infection [12]. NS4b contributes to viral replication by engaging with additional nonstructural proteins, changing host cell responses to promote viral transmission, and inducing autophagy to form viral replication complexes and evade host immune responses [10]. NS5 acts as the RNA-dependent RNA polymerase necessary for replicating the viral genome, with its N-terminal region involved in processes like viral RNA capping and evading the immune system by blocking interferon signaling and altering host cell transcriptional responses in DENV infection [13].

Understanding the immune correlates of protection is fundamental to rational vaccine design against DENV. Protective immunity against dengue is known to require a coordinated response involving both humoral and cellular arms of the adaptive immune system. Specifically, serotype-specific neutralizing antibodies—predominantly targeting the E protein domain III and the prM/E junction—are considered the primary correlates of protection [14]. CD4 ⁺ T helper cells amplify the antibody response and facilitate immunological memory, while CD8 ⁺ cytotoxic T lymphocytes (CTLs) eliminate virally infected cells and contribute to viral clearance [15]. However, the immunopathological phenomenon of antibody-dependent enhancement (ADE), in which heterologous, cross-reactive but non-neutralizing antibodies from a prior infection can facilitate uptake of DENV by Fcγ receptor-bearing cells, substantially complicates the requirement for balanced, broadly neutralizing immunity across all four serotypes [16]. Therefore, an effective dengue vaccine must elicit not only high-titer, serotype-spanning neutralizing antibodies but also robust and balanced T-cell responses, particularly CD4⁺ and CD8 ⁺ responses directed against conserved epitopes shared across serotypes.

The development of vaccines against DENV was initiated back in the 1920s but the dissimilitude among the four serotypes of DENV hindered the progress [17]. As of March 2024, only two dengue vaccines—Dengvaxia® and Qdenga®—are commercially available, and several vaccines are at different phases of clinical trials [18,19]. Despite being tetravalent, a major issue with Dengvaxia® is its efficacy is limited to only individuals ages 9−16 years who had been affected by DENV before getting vaccinated with this vaccine [20]. Dengvaxia® has also been reported to increase the risk of severe dengue in people having no prior dengue infections [20,21]. In contrast, Qdenga® is not limited to such conditions being applicable to anyone previously infected or not [22]. Another study shows that TAK-003—later known as Qdenga®—in phase 3 clinical trial, exhibited efficacy for long term and protection against all four DENV serotypes in individuals exposed to the virus previously [23]. DENV-1 and DENV-2 were successfully safeguarded by TAK-003 in dengue-naive individuals. This vaccine however, has not been approved yet by the Food and Drug Administration (FDA).

These limitations underscore the need for next-generation vaccine platforms that can safely and broadly engage the immune system across all four serotypes without the risks associated with live-attenuated approaches. Reverse vaccinology—the computational mining of pathogen genomes to identify antigenic targets—offers a systematic framework for identifying candidate epitopes with minimal risk of disease exacerbation [24]. Multi-epitope subunit vaccines designed through immunoinformatics eliminate live pathogenic components entirely, offer precise control over included antigens, and can be engineered to favor balanced humoral and cellular immune responses. Several prior immunoinformatics studies have proposed multi-epitope dengue vaccine candidates, providing a useful comparative framework. Kaushik et al. employed an immunoinformatics-based approach to design a conserved multi-epitope vaccine targeting all four dengue virus serotypes [25]. Their designed vaccine demonstrated favorable antigenic and immunogenic properties, stable interaction with immune receptor in molecular simulations, and successfully induced antibody production in an *in vivo* rabbit model, supporting its potential as a dengue vaccine candidate. Ishwar et al. report an *in silico* multi-epitope NS1/E-based vaccine candidate against DENV-1 and DENV-3 co-infection with favorable stability, strong immune and binding predictions, and successful codon optimization for experimental validation. [26]. Akash et al. propose a conserved epitope-based in-silico dengue vaccine targeting the envelope protein, showing strong antigenicity, TLR4 binding, and optimized expression with disulfide engineering, pending experimental validation [27]. Moreover, there are several prior studies on multi-epitope vaccines against dengue reported in the literature where researchers have tried to bring up a better solution against this disease [28]. Although a number of such designs have been reported, prior designed dengue vaccines report persistent gaps in all serotype coverage of dengue maintaining proper antigenicity and immune response. We focused on this gap for designing a multi-epitope vaccine which maintains covers all four serotypes of dengue virus, maintaining antigenicity, structural integrity, and proper immune response.

The present study addresses these gaps through a tetravalent multi-epitope vaccine designed by integrating B-cell and T-cell (CTL/MHC-I and HTL/MHC-II) epitopes derived from all four DENV serotypes, spanning both structural (C, prM, E) and non-structural (NS1–NS5) proteins. Unlike prior designs that focused on a single protein class or limited serotype coverage, this approach systematically evaluates the entire DENV proteome across all four serotypes and incorporates epitopes selected for high antigenicity, broad population coverage (based on HLA supertype distribution), and cross-serotype conservation. Human β-defensin 3 was incorporated as an N-terminal adjuvant to potentiate immune activation and enhance downstream adaptive responses. The assembled vaccine construct was evaluated for structural integrity, physicochemical properties, solubility, receptor binding affinity (via molecular docking with TLR2 and TLR4), and interaction stability (via molecular dynamics simulation). Immune simulations were performed to characterize the predicted humoral and cellular immune response profile, and *in silico* cloning was conducted to confirm expression feasibility. Taken together, this work presents a comprehensive computational framework for dengue vaccine design and provides a validated candidate for future experimental characterization.

## Methodology

### Viral protein sequence retrieval

**Fig 1:**
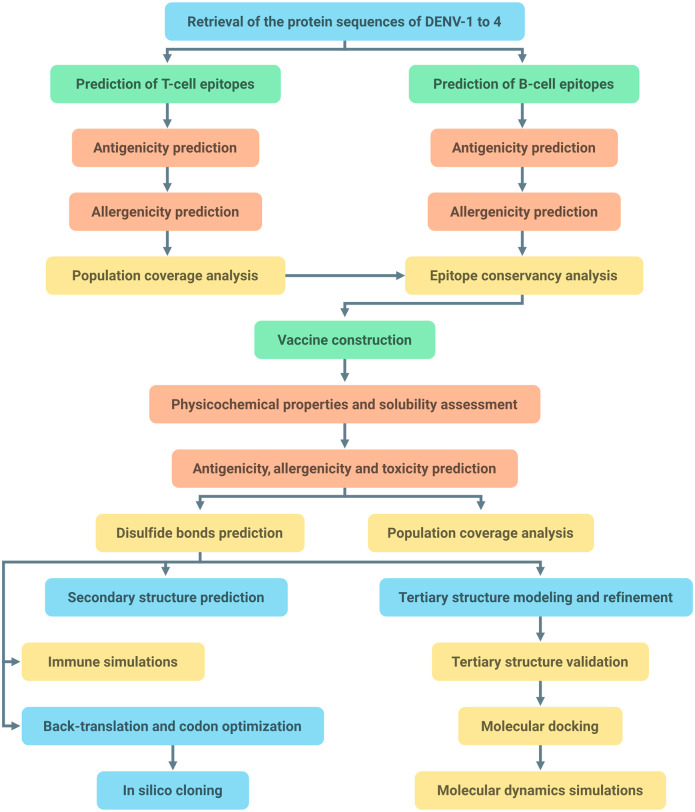
The workflow of the DENV vaccine design.

The initial step for designing the vaccine was the retrieval of the viral protein sequence of DENV-1 to −4 (Fig 1). The complete viral protein sequences of all dengue serotypes were selected for designing the tetravalent subunit vaccine. The polyprotein sequences from the complete genome of DENV-1 (Accession: NC_001477), DENV-2 (Accession: NC_001474), DENV-3 (Accession: NC_001475), and DENV-4 (Accession: NC_002640) were retrieved in FASTA format from the NCBI Genbank (https://www.ncbi.nlm.nih.gov/genbank/) [29]. Afterwards, the sequences were analyzed on the basis of all structural and functional proteins of dengue virus. The antigenicity of each protein of all sequences were determined from VaxiJen 2.0 server (https://www.ddg-pharmfac.net/vaxijen/VaxiJen/VaxiJen.html) [30]. When submitting to VaxiJen, the target organism was set to virus and the threshold value was set to 0.4. The antigenicity score was noted and compared for further analysis. It should be noted that these were the representative reference sequences for each serotype, which may not fully capture intra-serotype genetic diversity.

### B-cell epitopes prediction

B-cell epitopes were predicted from ABCpred (https://webs.iiitd.edu.in/raghava/abcpred/) [31]. The retrieved sequences for all serotypes in one letter-code without any header were pasted on this server individually. The prediction threshold value was set to 0.83, and the length of each epitope was fixed to 16 amino acids for each submission. Before submission of the sequences for prediction, the overlapping filter was activated. The ABCpred server employs an artificial neural network trained on linear B-cell epitopes obtained from the Bcipep database (https://webs.iiitd.edu.in/raghava/bcipep) for epitope prediction [31]. The maximum accuracy achieved was through a recurrent neural network (Jordan network) comprising a single hidden layer with 35 hidden units, using a window length of 16. Through fivefold cross-validation, the final network demonstrated an overall prediction accuracy of 65.93%, whereas the sensitivity, specificity, and positive prediction values were found to be 67.14%, 64.71%, and 65.61%, respectively [31].

### T-cell epitopes prediction

The cytotoxic T-lymphocyte (CTL) epitopes and helper T-lymphocyte (HTL) epitopes were predicted as MHC class I and class II binding epitopes, respectively, from the IEDB Immune Epitopes Database & Tools server (https://www.iedb.org/) [32]. During MHC-I binding prediction, the FASTA sequences were pasted individually and the prediction method was set to ANN 4.0. The whole HLA allele reference set was selected from the list. Afterwards, the epitopes were sorted based on IC_50_ values and epitopes having IC_50_ < 100 were prioritized for further analysis. In contrast, MHC-II binding epitopes were predicted using NN-align 2.3 (NetMHCII 2.3) prediction method for the full HLA reference set. The MHC-I binding epitopes were predicted in 9-meric form, whereas MHC-II epitopes were set to 15-mers.

### Antigenicity and allergenicity prediction of the epitopes

Antigenic property of the derived B-cell and T-cell epitopes were predicted using the VaxiJen v2.0 server [30]. The prediction of antigens in VaxiJen is alignment-free and relies on a range of physicochemical properties inherent to proteins [30]. While submitting each epitopes for prediction, the target organism was selected as virus and the threshold value was set to 0.4. All the epitopes surpassing the threshold value were considered as probable antigen and thus were subjected for allergenicity prediction. To predict whether these epitopes were non-allergen, AllerTOP v.2 server (https://www.ddg-pharmfac.net/AllerTOP/) was used [33]. The AllerTOP v.2 web server uses the auto cross covariance (ACC) method to convert protein sequences into uniform vectors. ACC, developed by Wold et al. (1993), employs five E descriptors from Venkatarajan and Braun (2001) to represent amino acid properties [34,35]. These descriptors include hydrophobicity, molecular size, helix-forming propensity, relative abundance, and β-strand forming propensity. Classification is done using k-nearest neighbor (kNN, k = 1) on a training set of 2427 known allergens and an equal number of non-allergens. Once the prediction for all epitopes were done, allergens were removed, and non-allergens were shortlisted.

### Conservancy and population coverage analysis of epitopes

The conservancy of each epitopes were analyzed using the Epitope Conservancy Analysis tool of IEDB Analysis Resource (http://tools.iedb.org/conservancy/) [32,36]. This analysis was performed to scrutinize how much the epitopes were conserved in the 4 serotypes of dengue. The threshold was set to a minimum of 80% while submission for analysis. Since the vaccine needs to be tetravalent to combat all serotypes, the epitopes conserved in the maximum number of serotypes were preferred. Afterwards, Population Coverage server of IEDB Analysis Resource (http://tools.iedb.org/population/) was utilized to detect the population coverage of each MHC-I and -II epitope classes [32]. The MHC-I and -II epitopes were uploaded to this server along with their respective alleles. The prediction was queried by “area_country_ethnicity” among the world population. The detailed tabulations from the server were analyzed for each MHC classes and epitopes were prioritized based on the worldwide coverage.

### Vaccine construction

An epitope-based subunit vaccine requires three types of elements—adjuvant, epitopes, and linkers—to elicit proper immune response as a complete vaccine. The vaccine sequence commences with the adjuvant when integrated into the primary peptide chain of the vaccine. The adjuvant plays a crucial role in stimulating the desired antigen-specific immune response, thereby enhancing the effectiveness of vaccination [37]. Human β-defensin 3 was selected as the adjuvant because of its potent immunomodulatory and antimicrobial properties, along with its reported ability to activate innate immune signaling pathways through interaction with Toll-like receptors (TLRs), particularly TLR1/TLR2 and TLR4 [38]. Previous studies have demonstrated that hBD-3 can induce dendritic cell maturation, promote chemotaxis of immune cells, stimulate cytokine production, and enhance adaptive immune responses through TLR-mediated signaling pathways [38]. The FASTA sequence of human β-defensin 3 (PDB ID: 1KJ6) was retrieved from the RCSB Protein Data Bank [39–41]. The adjuvant was added to the main vaccine sequence with an EAAAK linker. Subsequently, the MHC-I epitopes were included adjacent to the adjuvant and were connected to each other using AAY linkers. Next, the MHC-II epitopes were added, linked together by GPGPG linkers. Finally, the B-cell epitopes were incorporated into the sequence, with each epitope connected by KK linkers. This sequential arrangement ensures proper spacing and connectivity between the adjuvant, epitopes, and linkers, facilitating the induction of an effective immune response.

### Physicochemical properties and solubility assessment

The PepCheck (https://lab.sciencelet.org/pepcheck) and ExPASy ProtParam (https://web.expasy.org/protparam/) tools were employed to evaluate the physicochemical properties of the vaccine [42,43]. The amino acid sequence was entered into the server for assessment. Various parameters including the number of amino acids, molecular weight, molecular formula, theoretical isoelectric point (pI), amino acid compositions, extinction coefficients, estimated half-life, instability index, aliphatic index, and grand average of hydropathicity (GRAVY) values were analyzed in detail. Afterwards, the solubility of the vaccine construct was assessed and validated using three servers—SOLpro from SCRATCH Protein Predictor (https://scratch.proteomics.ics.uci.edu/) [44,45], SoluProt v1.0 [46], and Protein-Sol [47]. SOLpro utilizes a two-stage SVM architecture to predict the propensity of proteins to be soluble upon overexpression in *E. coli*. This method is based on multiple representations of the primary sequence [45]. Each classifier in the first layer takes a distinct set of features describing the sequence as input. Subsequently, a final SVM classifier aggregates the predictions from the first layer classifiers to predict whether the protein is soluble, along with providing a probability estimate. Similarly, SoluProt v1.0 also predicts the solubility of proteins upon expression in *E. coli* [46].

### Antigenicity, allergenicity and toxicity prediction of the vaccine

The antigenicity of the designed vaccine was predicted from the VaxiJen v2.0 and was validated using ANTIGENpro from SCRATCH Protein Predictor [45]. The FASTA sequence of the vaccine construct was uploaded to VaxiJen. The target organism was chosen as virus keeping the threshold value a minimum of 0.4. After retrieval of the antigenicity from VaxiJen, it was further validated from the ANTIGENpro tool. Consequently, the vaccine was subjected to allergenicity and toxicity prediction. The AllerTOP v.2 server was utilized to predict whether the vaccine construct is non-allergenic. Afterwards, the FASTA format of the sequence was submitted to the T3DB server (http://www.t3db.ca/) for toxicity prediction [48]. In this process, the costs to open and extend a gap were set to −1, the penalty for mismatch was set to −3, and the reward for match was set to 1. The expectation value was set to 0.00001. Gapped alignment was performed, and the query sequence was filtered by checking the respective checkboxes during sequence submission. Finally, by conducting the search, the toxicity of the vaccine was predicted.

### Population coverage analysis of the vaccine

IEDB Population Coverage server was again used at this step to assess the population coverage of the vaccine construct [32]. The two epitope classes (MHC-I and MHC-II) were uploaded with their respective alleles as done before. The prediction was queried also as before by selecting “area_country_ethnicity” among the all population of the world, also separately for East Asia, Northeast Asia, South Asia, Southeast Asia, Southwest Asia, Europe, East Africa, West Africa, Central Africa, North Africa, South Africa, West Indies, North America, Central America, South America, and Oceania. These areas were chosen because these were marked as the major regions of the world as per the IEDB Population Coverage website. The calculation was conducted separately for each MHC class and combined. The coverage, average hit, and PC90 values were scrutinized. The standard deviation was also taken into account while analyzing.

### Disulfide bonds prediction

The prediction of disulfide bonds in the vaccine construct was performed using the DIpro tool of the SCRATCH Protein Predictor server. DIpro is a cysteine disulfide bond predictor that utilizes a 2D recurrent neural network. It supports various algorithms including SVM, graph matching, and regression [49]. This tool is capable of predicting whether a peptide sequence contains disulfide bonds, estimating the number of disulfide bonds present, and predicting the bonding state of each cysteine and the bonded pairs. Upon submission, the peptide sequence undergoes processing in two steps [49]. Firstly, DIpro employs SVM to classify whether the sequence contains disulfide bonds. Subsequently, it utilizes neural network and graph algorithms to predict the number of disulfide bonds and the bond pattern.

### Secondary structure prediction

Secondary structure of the vaccine was predicted from the PSIPRED 4.0 server (http://bioinf.cs.ucl.ac.uk/psipred/) [50]. Upon submission of the protein sequence, PSIPRED generated three types of sequence plots and a cartoon. The figures were analyzed to predict the structure of different portions in the vaccine peptide chain as strand, helix, or coil. The type of amino acids—small nonpolar, hydrophobic, polar, or aromatics plus cystiene—were also obtained from a plot. Moreover, from the cartoon, the structures were obtained with different confidence levels of prediction.

### Tertiary structure modeling and refinement

The tertiary structure of the vaccine was predicted using the 3Dpro tool of the SCRATCH Protein Predictor server. The peptide sequence of the vaccine was pasted to the server and submitted for the prediction. 3Dpro utilizes predicted structural features and PDB knowledge-based statistical terms to construct multiple peptide models using random seeds. The model with the lowest energy is selected as the final prediction. Notably, this method is currently a de novo approach, meaning it does not rely on structural templates. Hence, the PDB file of the vaccine 3D model was retrieved from 3Dpro results. Following the initial modeling, the structure was refined using the GalaxyRefine tool from the GalaxyWEB server (https://galaxy.seoklab.org/cgi-bin/submit.cgi?type=REFINE) [51–53]. The PDB file of the tertiary structure was uploaded and submitted for refinement. From the refinement results, the GDT-HA, MolProbity, and Rama favored scores were obtained. The model with the highest Rama favored score was chosen for further validation. The graphical image of the epitopes in the tertiary structure showing surface accessibility, was generated in PyMOL software [54].

### Tertiary structure validation

The Ramachandran plot of the peptide sequence was analyzed using the PROCHECK tool of the SAVES v6.0 structure validation server (https://saves.mbi.ucla.edu/) [55]. The tertiary refined model was uploaded to the server, and the PROCHECK tool was executed. The Ramachandran plot provided information on the allowed regions of each amino acid residue, aiding in the interpretation of the structural quality. Additionally, chi1-chi2 plots, main-chain and side-chain parameters, residue properties, G-factors, and planar groups were analyzed to further assess the structural properties of the model. Afterwards, the Z-score of the peptide model was evaluated using ProSA-web (https://prosa.services.came.sbg.ac.at/prosa.php) [56]. The Z-score provides an indication of the overall quality and compatibility of the model with experimental data and serves as a measure of its structural integrity (Wiederstein and Sippl, 2007).

### Molecular docking of the vaccine with immune receptors

Molecular docking was carried out to investigate the interaction between the vaccine construct and immune receptors in the human body. The docking tasks were performed using the ClusPro 2.0 server (https://cluspro.bu.edu/) [57–60]. The vaccine model was docked separately with TLR2 (PDB ID: 2Z7X) and TLR4 (PDB ID: 3FXI), whose PDB structures were obtained from the RCSB PDB server [61,62]. ClusPro 2.0 automatically detected the binding pockets of the immune receptors and docked the vaccine to the specific suitable site of the receptors. To ensure efficient processing, both the CPU and GPU servers of ClusPro were utilized to run and validate the docking results. ClusPro employs FFT-based global sampling using PIPER to explore docking orientations efficiently, followed by clustering to identify low-energy conformations [59]. It then utilizes CHARMM minimization to refine the structures, removing steric clashes. The interactions of the docked complexes were scrutinized using the PDBsum tool (https://www.ebi.ac.uk/thornton-srv/databases/pdbsum/) [63]. The clusters were further analyzed in HADDOCK 2.4 (https://wenmr.science.uu.nl/haddock2.4/) and COCOMAPS 2.0 (https://aocdweb.com/BioTools/cocomaps2/) for van dar Waals energy, electrostatic energy, buried surface area, and interface stability [64–65].

### Molecular dynamics simulations

Molecular dynamics is a sophisticated automated simulation approach used for evaluating the degree of stability of protein and protein-ligand complex structure at the minuscule stage through demonstrating the behavioral characteristics, interacting arrangement, fluctuation, the physical foundation of function, and structure. The molecular dynamics (MD) simulations were conducted using GROMACS version 2023.1 to investigate the behavior of both vaccine–TLR4 complex and vaccine–TLR2 complex [66]. The protein was parameterized for its protein content using the CHARMM General Force Field. The SwissParam server was utilized to perform the ligand topologies [67]. The structures underwent 2500 cycles of vacuum minimization using the steepest descent method in order to mitigate any potential steric issues. The solvation of the structure was accomplished through the utilization of the Simple Point Charge (SPC) water model. Subsequently, the system was rendered neutral by introducing Na^+^ and Cl^-^ ions utilizing the *gmx genion* tool. This measure was implemented in order to maintain the overall electrical neutrality of the system. After minimization, three steps were conducted in the MD simulation: NVT, NPT, and production. The equilibration of the systems was conducted in two phases. Initially, an NVT equilibration lasting 100 picoseconds was conducted to attain a steady state of the number of particles, volume, and temperature. The purpose of this step was to elevate the system to a temperature of 300 Kelvin. The second step involved conducting a 100 picoseconds NPT equilibration, which aimed to maintain the system’s pressure and density stability by ensuring an equal number of particles, pressure, and temperature. The production MD simulations were carried out under the NPT ensemble. The simulations involved the imposition of bond constraints on all bonds within the protein, thereby inducing position restraint of the protein group. The constrained conditions of NVT and NPT resulted in the relaxation of water molecules surrounding the protein, leading to a decrease in system entropy. Temperature coupling was controlled using the modified Berendsen velocity-rescaling (v-rescale) thermostat and pressure coupling was maintained using the Parrinello–Rahman barostat [68]. The relaxation of the barostat and thermostat persisted for a duration of 100 picoseconds. The application of Linear Constraint Solver algorithm was utilized to impose constraints on the covalent bonds. The Particle-Mesh Ewald (PME) method was employed to process the electrostatic interactions. After reaching equilibrium, every system underwent a production run that lasted for 200 nanoseconds (ns) of simulation time. A timestep of 2 fs was used throughout the equilibration and production simulations.

### Immune simulations

Immune simulations were run to scrutinize the immune response inside the body. C-ImmSim server (https://kraken.iac.rm.cnr.it/C-IMMSIM/) was used to run the immune simulations [69–71]. The simulations were run under a random seed. The simulation volume was set to 10 and the simulation was run for 1000 steps (for around 333 days). The host HLA selections were chosen for A0101, A0201, B0702, B0801, DRB1_0101, and DRB3_0101 respectively. Total 3 injections were included at the interval of 20 days. The time steps with this interval represents 1, 61, and 121, respectively, for the 1st, 21st, and 41st days. The number of antigens for each dose was set to 1000. The FASTA sequence of the vaccine was inputted as the Ag molecule. After setting up the pre-submission parameters, the vaccine was submitted for the simulations.

### Back-translation, optimization and *in silico* cloning

Back-translation of the vaccine peptide sequence was conducted using the EMBOSS Backtranseq server (https://www.ebi.ac.uk/Tools/st/emboss_backtranseq/), selecting the *Esh coli* K12 strain for codon usage [72]. The resulting back-translated sequence was retrieved from the server. Later on, Java Codon Adaptation Tool (JCAT; https://www.jcat.de/) was utilized for codon optimization, with the organism set as *E. coli* (strain K12) [73]. During optimization, avoidance of rho-independent transcription terminators, prokaryotic ribosome binding sites, and *Eco*RI restriction enzyme cleavage sites was ensured. The codon adaptation index (CAI) and the GC-content of the improved sequence were recorded. A higher CAI value (0.8–1.0) is always expected as higher CAI indicates higher expression [74]. Afterwards, *in silico* restriction cloning of the vaccine was performed using SnapGene software (www.snapgene.com), employing the *E. coli* pET-28a(+) vector. Prior to cloning, *Nde*I and *Xba*I restriction enzyme sites were incorporated at the N- and C-terminals of the optimized DNA sequence. Finally, the DNA sequence was inserted into the pET-28a(+) vector.

## Results

### Viral protein sequence

The viral polyprotein sequences of DENV-1, DENV-2, DENV-3, and DENV-4 were 3392, 3391, 3390, and 3387 amino acids in lengths, respectively (S1 Table). The polyprotein comprises of structural and non-structural proteins. The structural proteins of the virus play a pivotal role in facilitating host invasion and orchestrating the intricate assembly of viral particles. In contrast, non-structural proteins assume a key function in the viral life cycle, secreting diverse enzymes that actively contribute to the intricate processes of viral replication and the synthesis of structural proteins. The interplay between these structural and non-structural components are crucial in the viral life cycle.

### B-cell epitopes prediction and prioritization

B-cell epitopes were predicted for the complete sequence of all DENV serotypes utilizing the ABCPred algorithm. The shortlisting of epitopes hinged upon a stringent criterion, incorporating a prediction score ≥ 0.83, alongside considerations of antigenicity, allergenicity, and toxicity. Only probable antigens and probable non-allergens were sorted out for further progress. The length of each B-cell epitopes were 16 amino acids as specified while submitting the sequence for the prediction. Moreover, the B-cell epitopes that were found in the maximum serotypes of DENV were favored. Among the selected epitopes, DENV-2 was represented by only one B-cell epitope, whereas all other serotypes had 2 epitopes for each, of which some are common. The finally selected B-cell epitopes are presented in Table 1.

**Table 1:** Prioritized B-cell epitopes, their antigenicity, and conservancy across different DENV serotypes.

B-cell Epitopes	Antigenicity Score	DENV-1	DENV-2	DENV-3	DENV-4
RNLTIMDLHPGSGKTR	1.5229	✓		✓	
TAGWDTRITEDDLQNE	0.7913	✓			✓
APSYGMRCVGVGNRDF	1.9706		✓		
KTKKDLGLGSIATQQP	1.4308			✓	
TGEIGAIALDFKPGTS	1.7277				✓

### T-cell epitopes prediction and prioritization

T-cell epitopes, crucial for immune response, were predicted from the IEDB T-cell epitope prediction tools, discerning between MHC-I (9-mer) and MHC-II (15-mer) epitopes. Several factors were assessed while prioritizing the epitopes: combined score, reflecting overall epitope quality; antigenicity, indicating the likelihood of eliciting an immune response; allergenicity, to avoid potential allergic reactions; and IC_50_ values below 100 nM, denoting strong binding affinity to MHC molecules. The epitopes were then subjected to antigenicity and allergenicity prediction, and the epitopes with higher antigenicity scores and probable non-allergenic properties were prioritized. Moreover, epitope conservancy across DENV serotypes was scrutinized for maximum DENV serotypes coverage. For ensuring common antigenic regions across different DENV strains, epitopes shared among all or most serotypes were kept at top priority. The finally selected T-cell epitopes of both MHC-I and -II classes are shown in Table 2.

**Table 2:** Prioritized T-cell epitopes, their antigenicity, and conservancy across different DENV serotypes. The epitopes are shown based on classes (MHC-I and -II).

Epitope class	Epitope	Antigenicity Score	DENV-1	DENV-2	DENV-3	DENV-4
MHC-I	TVWFVPSIK	0.8732	✓	✓	✓	✓
MHC-I	YFHRRDLRL	1.6075	✓	✓	✓	✓
MHC-I	KTRTNDWDF	2.3435		✓		
MHC-I	IAASIILEF	0.4992	✓	✓		✓
MHC-I	ETACLGKSY	0.8757	✓	✓		
MHC-I	REWCFTGER	2.2702				✓
MHC-I	ASAAQRRGR	1.442	✓		✓	✓
MHC-I	LEFEALGFL	1.9477		✓	✓	✓
MHC-I	GLNSRSTSL	1.8196	✓			
MHC-I	RTTWSIHAK	1.6336		✓		
MHC-I	FTMGVLCLA	1.7481			✓	
MHC-I	DIISRKDQR	1.5041			✓	✓
MHC-II	AKGSRAIWYMWLGAR	0.6958	✓	✓	✓	✓
MHC-II	GQVGTYGLNTFTNME	0.6755	✓	✓		✓
MHC-II	MYADDTAGWDTRITE	0.6312	✓		✓	✓
MHC-II	FHRRDLRLAANAICS	1.0313	✓	✓		
MHC-II	KKVIQLSRKTFDTEY	0.6002			✓	✓
MHC-II	LHPASAWTLYAVATT	0.6629	✓		✓	

### Vaccine construct

**Fig 2:**
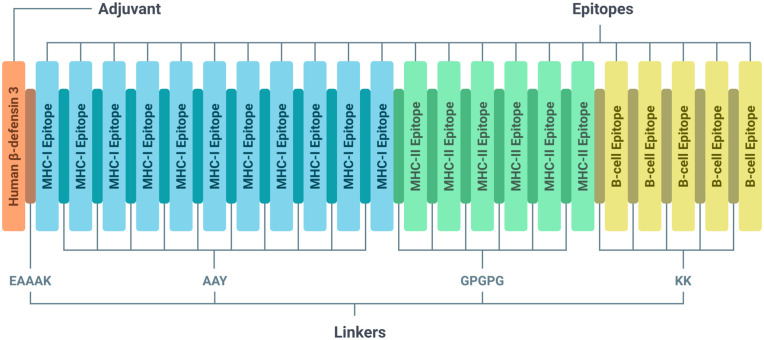
The vaccine construct. The adjuvant, 3 types of epitopes, and 4 types of linkers are shown in different colors.

The final vaccine exhibited a length of 401 amino acids. The N-terminal residue of the construct was Glycine (Gly). The adjuvant human β-defensin 3 was linked to the core vaccine sequence with an EAAAK linker. The vaccine construct encompassed a total of 23 epitopes, comprising 12 MHC-I epitopes, 6 MHC-II epitopes, and 5 B-cell epitopes (Fig 2). Among these, DENV-1, DENV-2, DENV-3, and DENV-4 were represented by 13, 11, 12, and 13 epitopes, respectively. Moreover, among the 12 MHC-I epitopes, 2 were found common across all DENV serotypes. To be more specific, 6 MHC-I epitopes were attributed to DENV-1, 7 to DENV-2, 6 to DENV-3, and 7 to DENV-4. In contrast, among the MHC-II epitopes, DENV-1, DENV-2, DENV-3, and DENV-4 were specific to 5, 3, 4, and 4 epitopes, respectively, with a subset being common across the serotypes.

### Physicochemical properties and solubility assessment

Physicochemical properties of the construct were assessed from PepCheck (https://lab.sciencelet.org/pepcheck) and thus were validated using ExPASy ProtParam tool. The molecular formula of the vaccine was determined as C_1950_H_3053_N_525_O_555_S_17_ and the molecular weight stood at 43.84 kDa. The major parameters, including molecular weight, atom count, total negatively charged residues (Asp + Glu), total positively charged residues (Arg + Lys), and isoelectric point (pI), fell within optimal ranges (Table 3). The stability assessment, characterized by an instability index below 40, indicated the vaccine’s robust nature. The vaccine, with a GRAVY value below 0.00, exhibited hydrophilic properties. Solubility predictions from SOLpro, SoluProt 1.0, and Protein-Sol scored 0.823, 0.794, and 0.519, respectively. The scores affirmed the soluble nature of the vaccine construct.

**Table 3:** Physicochemical properties and solubility of the vaccine construct.

Parameters	Vaccine construct measures
Molecular weight	43.84 kDa
Number of atoms	6140
Number of amino acids	401
Total number of negatively charged residues (Asp + Glu)	32
Total number of positively charged residues (Arg + Lys)	63
Theoretical isoelectric point (pI)	9.81
Extinction coefficient	81415 M^-1 cm-1^
Instability index	28.06
Estimated half life	30 hours (mammalian reticulocytes, *in vitro*)
	>20 hours (yeast, *in vivo*)
	>10 hours (*Escherichia coli*, *in vivo*)
Aliphatic index	64.94
Grand average of hydropathicity (GRAVY)	−0.444
Solubility	0.823 (SOLpro)
	0.794 (SoluProt 1.0)
	0.519 (Protein-Sol)

### Antigenicity, allergenicity and toxicity of the vaccine

The antigenicity of the vaccine was 0.9319 as per VaxiJen 2.0 which is significantly higher than the threshold 0.4. The antigenicity was verified using ANTIGENpro server and it was 0.813649. Afterwards, on assessment of the allergenicity of the vaccine, the vaccine was found to be probable non-allergen as per AllerTOP v.2 and AlgPred server. AlgPred showed a score of −0.63394281 for the vaccine candidate, that surpasses the threshold of maximum −0.40 to be non-allergen. Finally, on submission of the FASTA sequence of the vaccine to the T3DB server, no results came from the database, thus indicating the vaccine as non-toxic.

### Population coverage

**Fig 3:**
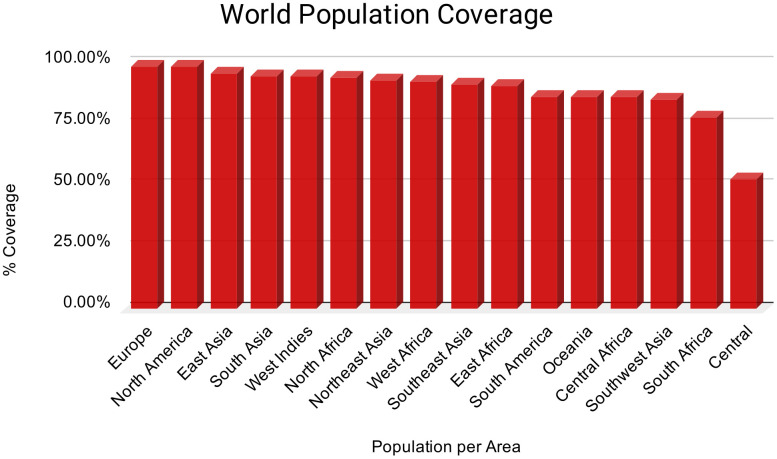
Worldwide population coverage chart of the designed vaccine. Each region shows the percentage of coverage as per the data retrieved from the IEDB Population tool.

The worldwide population coverage of the vaccine was 97.35% as per IEDB Population Coverage server (Fig 3 and S1 Fig). The predicted population coverage was highest in Europe (98.56%) and North America (98.56%), followed by East Asia (95.95%), South Asia (95.06%), the West Indies (94.83%), North Africa (94.30%), Northeast Asia (93.18%), West Africa (92.43%), Southeast Asia (91.53%), East Africa (91.09%), South America (86.59%), Oceania (86.54%), Central Africa (86.32%), Southwest Asia (85.42%), South Africa (78.14%), and Central America (52.76%), as illustrated in Fig 3. Such extensive coverage indicates the potential for widespread immunization and highlights the vaccine’s substantial impact in combating the targeted pathogen. The average hit combining both MHC-I and -II epitopes was 3.53 with a PC90 of 1.74 (S2 Table). For MHC-I, 85.42% population coverage was obtained with average hits of 2.02 and PC90 of 0.69. In contrast, for MHC-II, the population coverage was 81.81% with average hits of 1.50 and PC90 of 0.55. The average hits are the average number of epitope hits or, HLA combinations recognized by the population, whereas the PC90 value is the minimum number of epitope hits or, HLA combinations recognized by 90% of the population.

### Disulfide bonds analysis

DIpro tool of SCRATCH Protein Predictor predicted that the sequence does have disulfide bonds. This tool detected 11 cysteines in the amino acid chain and predicted 4 disulfide bonds that may form in the sequence. Specifically, cysteines at positions 11, 18, 33, 40, 102, 114, 270, and 357 are anticipated to participate in the formation of these disulfide bonds. The classification results were obtained using Support Vector Machine (SVM). The predicted bonds (cysteine pairs) are listed in Table 4, ordered by probability in descending order.

**Table 4:** Predicted disulfide bonds (cysteine pairs). The bonds shown in the table are ordered by probability in descending order.

Bond Index	Cys1 Position	Cys2 Position
1	11	18
2	270	357
3	102	114
4	33	40

### Secondary structure

**Fig 4:**
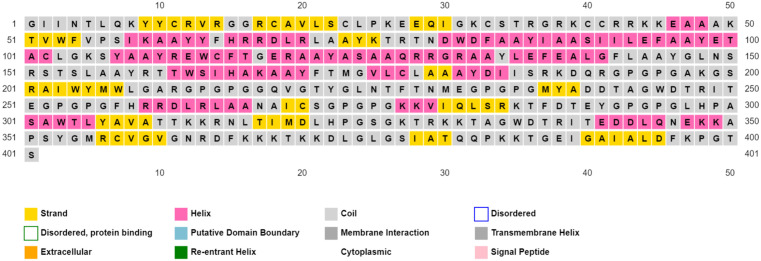
Predicted secondary structure. Different portions are colored based on the predicted structure.

The PSIPRED 4.0 server generated the secondary structure prediction for the vaccine peptide based on the submitted amino acid sequence. In the provided output, pink regions represent helices, yellow regions indicate strands, and gray regions resemble coils (Fig 4). Additionally, the confidence levels are denoted by the darkness of the color bars, with darker bars indicating higher confidence compared to lighter bars (S2 Fig). Among 401 amino acids, 63 (15.71%) formed strand, 112 (27.93%) involved in helix formation, and the rest (226, 56.36%) represented coil structures.

### Tertiary structure modeling and refinement

**Fig 5:**
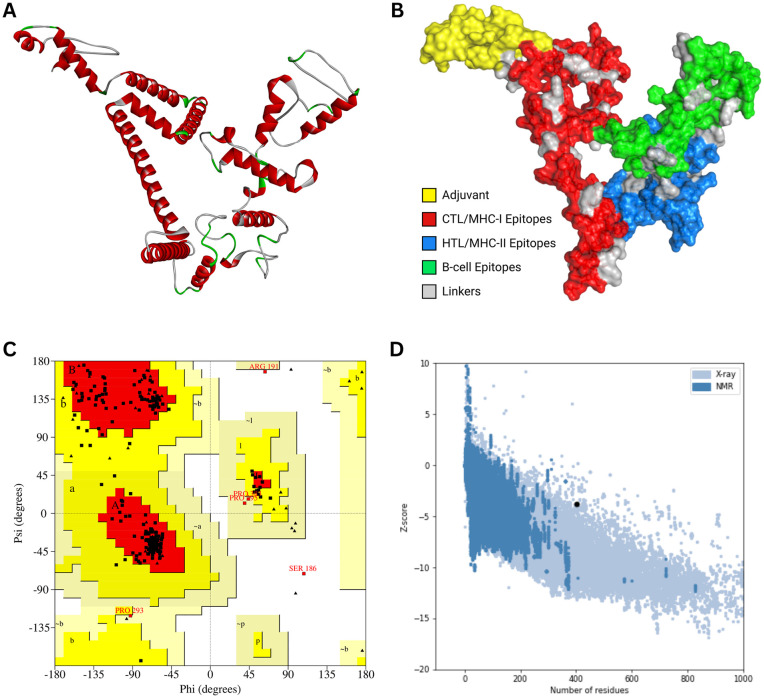
Prediction and validation of tertiary structure. (A) Refined tertiary structure of the vaccine. (B) Surface exposure of the epitopes. (C) Ramachandran plots. (D) Z-score plot.

The tertiary structure of the protein in PDB format was downloaded from the 3Dpro tool of SCRATCH Protein Predictor. Upon prediction, the structure was refined using the GalaxyRefine tool, which yielded five polished models. Among the refined models, model 4 was selected due to its favorable Rama score of 95.6. Additionally, the GDT-HA score for model 4 was determined to be 0.8953. Moreover, the model has a root mean square deviation (RMSD) of 0.559 and a MolProbity of 2.011. The refined tertiary structure of the vaccine is shown in Fig 5A. Moreover, the surface accessibility of the epitopes in the vaccine are shown in Fig 5B.

### Tertiary structure validation

The PROCHECK v.3.5 tool of SAVES v6.0 server analyzed the refined structure and illustrated a Ramachandran plot (Fig 5C). The Ramachandran plot depicts that 93.1% (312) of residues were found in the most favored region and 6.3% (21) occupied the additionally allowed region among the considerable 335 (of 401) residues. Notably, no residues were observed in the generously allowed regions and only 0.6% (2) residues were in the disallowed regions, indicating the structural integrity of the peptide. The residues SER186 and ARG191 are the residues in the unfavored or disallowed region in the Ramachandan plot. Subsequently, the vaccine construct yielded a Z-score of −3.83 which was retrieved from ProSA-web server (Fig 5D).

### Molecular docking analysis

**Fig 6:**
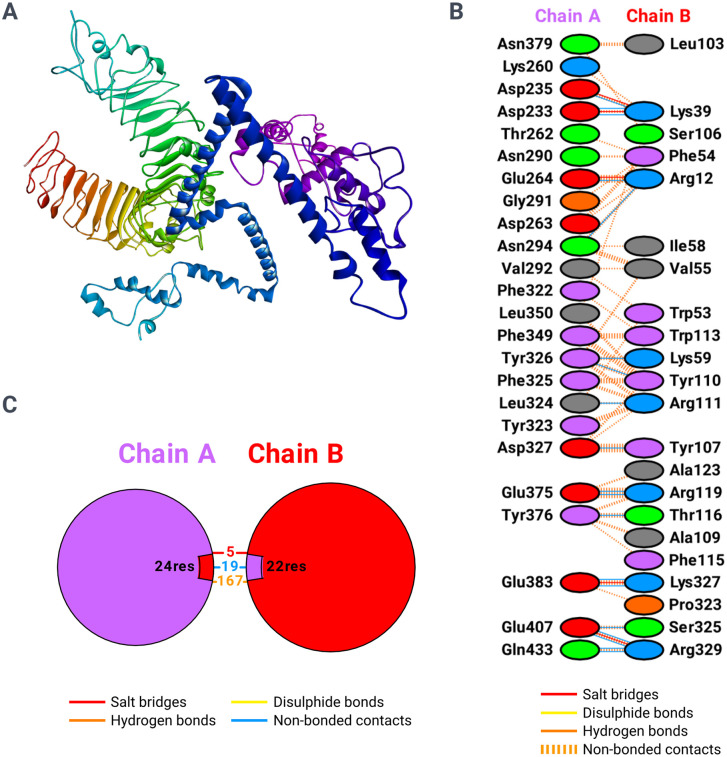
Molecular docking of the vaccine (chain B) with TLR2 (chain A). (A) The three dimensional conformation of vaccine–TLR2 complex. (B) Interactions between the vaccine and TLR2 characterized by types and amino acids. (C) Overall total number of interactions between the vaccine and TLR2 by types. (D) Electrostatic energy of the docked cluster. (E) van dar Waals energy of the docked cluster.

**Fig 7:**
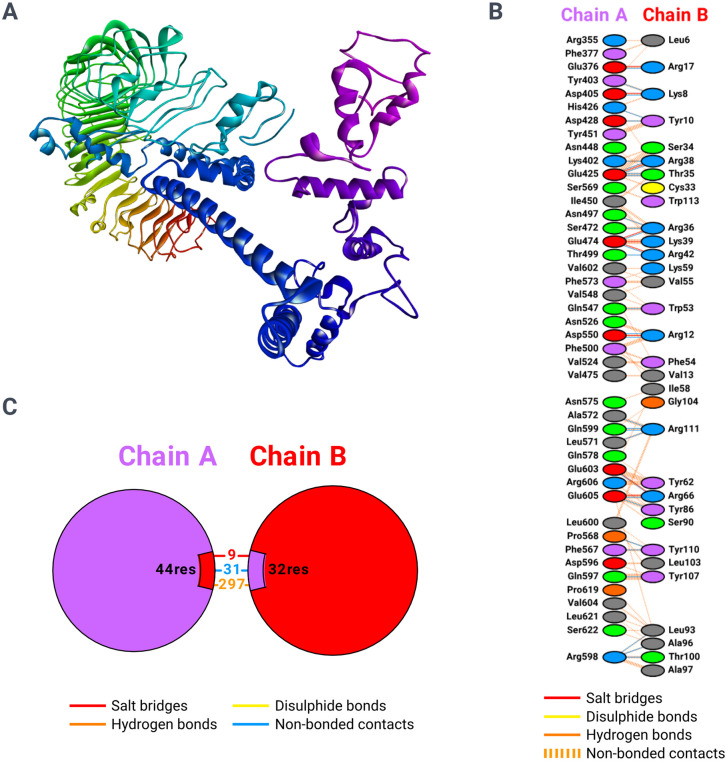
Molecular docking of the vaccine (chain B) with TLR4 (chain A). (A) The three dimensional conformation of vaccine–TLR4 complex. (B) Interactions between the vaccine and TLR4 characterized by types and amino acids. (C) Overall total number of interactions between the vaccine and TLR4 by types. (D) Electrostatic energy of the docked cluster. (E) van dar Waals energy of the docked cluster.

Once the molecular docking analyses were performed using the ClusPro 2.0 server’s supercomputers to investigate the interactions between the vaccine model and toll-like receptor 2 (TLR2) and TLR4, the vaccine demonstrated significant interactions with both the immune receptors. The docking scores reported by ClusPro correspond to clustering-based energy functions used to rank predicted complexes and do not represent absolute binding free energies. A total of 30 docked conformations were yielded in the docking operations. The top results had been considered for each operation. The complex formed with TLR2 and TLR4 had a lowest cluster-weighted energy scores of −1393.3 kJ/mol and −1240.5 kJ/mol, respectively; these values reflect relative docking favorability within the ClusPro scoring framework rather than absolute thermodynamic binding energies. The docked complexes with TLR2 and TLR4 have been shown in Figs 6 and 7. A total of 22 residues were observed from the vaccine and that of 24 residues from TLR2 were observed participating in different types of interactions (Fig 6B and 6C). Specifically, 5 salt bridges, 19 non-bonded contacts, and 167 hydrogen bonds were observed in the vaccine–TLR2 complex (Fig 6C). In contrast, in the vaccine–TLR4 complex, 44 residues from the vaccine and 32 residues from TLR4 were speculated to interact (Fig 7B and 7C). In this complex, 9 salt bridges, 31 non-bonded contacts, and 297 hydrogen bonds were formed (Fig 7C).

To further characterize the stability and driving forces of the vaccine-receptor interface, the docked complexes were refined using the HADDOCK 2.4 server. This analysis provided a quantitative breakdown of the electrostatic and Van der Waals (vdW) contributions to the binding affinity (Table 5). The vaccine–TLR2 complex (Cluster 1) exhibited a HADDOCK score of −208.3 ± 2.4. This interaction was supported by a Van der Waals energy of −83.2 ± 3.3 kcal/mol and a significant electrostatic energy of −477.6 ± 17.1 kcal/mol. The buried surface area (BSA) for this interaction was calculated at 2754.0 ± 57.5 A2, indicating a broad and stable interface.

**Table 5:** HADDOCK refinement energetics of vaccine–receptor complexes. Refinement parameters obtained from HADDOCK for the vaccine complexes with TLR2 and TLR4. The evaluated parameters include HADDOCK score, van der Waals energy, electrostatic energy, desolvation energy, and buried surface area (BSA). More negative HADDOCK, van der Waals, and electrostatic energy values indicate stronger and more favorable intermolecular interactions, whereas larger buried surface area values reflect greater interface contact and complex stability.

Parameter	Vaccine–TLR2 Complex	Vaccine–TLR4 Complex
**HADDOCK Score**	−208.3 ± 2.4	−293.3 ± 7.5
**Van der Waals Energy (kcal/mol)**	−83.2 ± 3.3	−106.2 ± 7.1
**Electrostatic Energy (kcal/mol)**	−477.6 ± 17.1	−828.1 ± 20.6
**Desolvation Energy (kcal/mol)**	−29.6 ± 2.3	−21.5 ± 3.7
**Buried Surface Area (Å**^**2**^)	2754.0 ± 57.5	3860.5 ± 49.1

For vaccine–TLR4 complex (Cluster 1), the interaction demonstrated even higher energetic favorability with a HADDOCK score of −293.3 ± 7.5. This complex was stabilized by a Van der Waals energy of −106.2 ± 7.1 kcal/mol and a dominant electrostatic contribution of −828.1 ± 20.6 kcal/mol. The extensive BSA of 3860.5 ± 49.1 A2 further corroborates the formation of a highly stable complex between the vaccine and TLR4. The strongly negative electrostatic scores represent the primary driving force for the binding, likely mediated by the numerous salt bridges and hydrogen bonds previously identified. The negative Van der Waals values reflect the favorable shape complementarity and close atomic packing at the receptor-ligand interface, which are essential for effective immune recognition.

**Fig 8:**
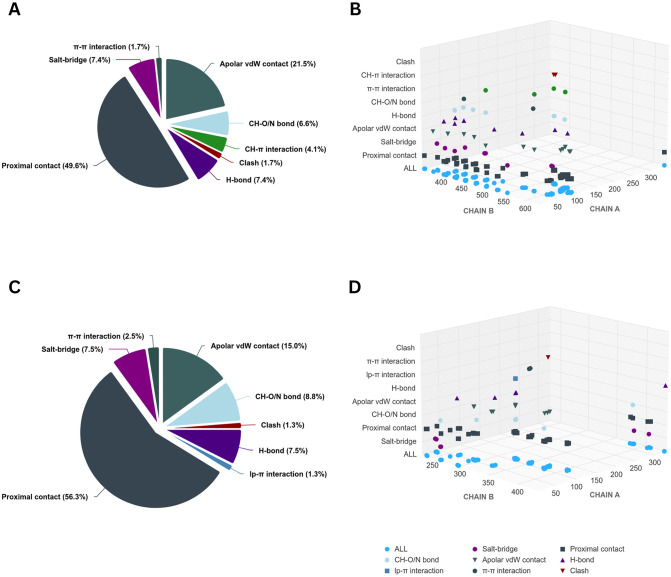
Interaction profiling and docking energetics of vaccine–TLR complexes. (A) Distribution of intermolecular interactions in the vaccine–TLR2 complex. (B) Three-dimensional interaction mapping of residues involved in the vaccine–TLR2 complex. (C) Distribution of intermolecular interactions in the vaccine–TLR4 complex. (D) Three-dimensional interaction mapping of residues involved in the vaccine–TLR4 complex. Interaction analyses include proximal contacts, hydrogen bonds, salt bridges, apolar van der Waals contacts, π–π interactions, CH–π interactions, lp–π interactions, and steric clashes. Docking refinement energetics obtained from HADDOCK demonstrated favorable binding stability for both complexes, with the vaccine–TLR4 complex exhibiting comparatively stronger interaction energetics and larger buried surface area than the vaccine–TLR2 complex.

Fig 8 illustrates intermolecular interaction profiling of the vaccine construct with TLR2 (A–B) and TLR4 (C–D). The vaccine–TLR2 complex demonstrates diverse binding interactions dominated by proximal contacts (49.6%) and apolar van der Waals contacts (21.5%), indicating extensive interface complementarity and hydrophobic stabilization. Hydrogen bonds, salt bridges, and CH interactions further contribute to binding stability. Conversely, the vaccine–TLR4 complex exhibits a higher proportion of proximal contacts (56.3%) but comparatively fewer hydrophobic interactions (15.0%). Additional lp–π and π–π interactions suggest electronic stabilization within the binding interface. Overall, both complexes display stable and favorable interaction networks, with TLR2 showing relatively stronger hydrophobic interaction contributions.

### Molecular dynamics (MD) simulation analysis

**Fig 9:**
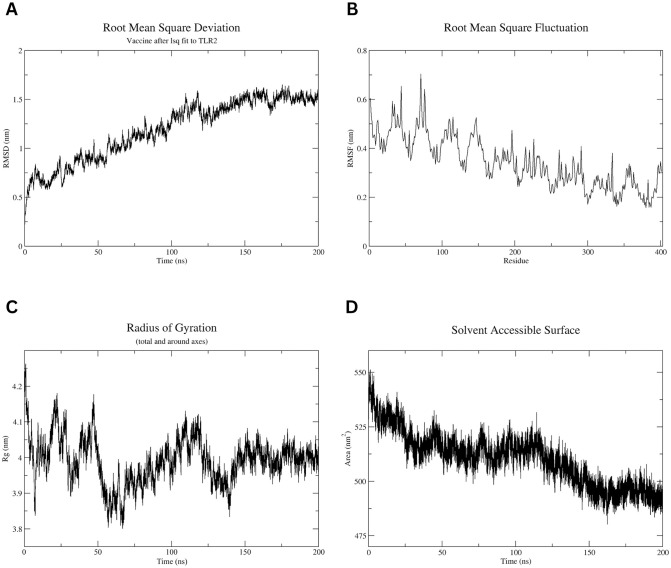
Molecular dynamics simulation graphs of the vaccine with TLR2. (A) Root mean square deviations (RMSD). (B) Root mean square fluctuations (RMSF). (C) Radii of gyration (Rg). (D) Solvent accessible surface area (SASA).

**Fig 10:**
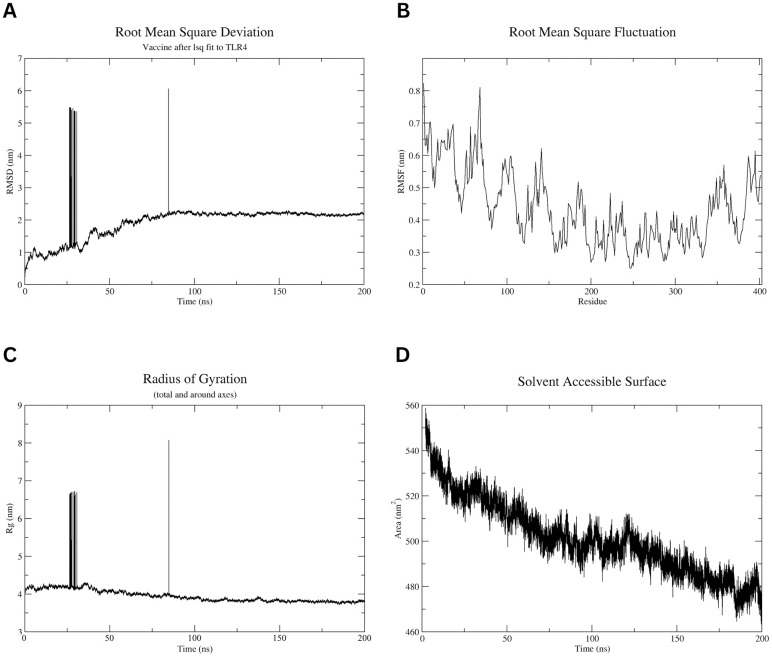
Molecular dynamics simulation graphs of the vaccine with TLR4. (A) Root mean square deviations (RMSD). (B) Root mean square fluctuations (RMSF). (C) Radii of gyration (Rg). (D) Solvent accessible surface area (SASA).

The structural integrity and conformational stability of the vaccine–TLR2 complex were evaluated through a comprehensive MD simulation, with parameters analyzed via the XmGrace package (Fig 9 and 10) [73]. The root mean square deviation (RMSD), which monitors time-dependent structural shifts from the initial state [74,75], yielded an average value of 1.187 nm. Stability was particularly evident between 16 and 22 ns (Fig 9A), where the RMSD reached a plateau, suggesting the complex fluctuated around a consistent, stable average conformation. Local residue flexibility was assessed using root mean square fluctuation (RMSF) [76]. The complex displayed constrained movements, with an average RMSF of 0.301 nm; fluctuation was less among the residues with later positions (Fig 9B). Furthermore, the radius of gyration (RG), which characterizes the mass-weighted root mean square distance of atoms from their common center of mass [76], remained stable between 150 and 165 ns (Fig 9C) with an average value of 3.99 nm. Finally, the solvent accessible surface area (SASA) analysis, indicative of the protein’s interactive potential with the aqueous environment [76], showed a stable range from 50 to 110 ns with an average area of 510.15 nm^2^ (Fig 9D). These metrics collectively suggest that the vaccine–TLR2 complex maintains a high degree of structural stability and surface accessibility throughout the simulation.

The dynamic behavior of the vaccine–TLR4 complex was characterized to determine its stability under simulated physiological conditions. The RMSD analysis revealed an average value of 1.913 nm (Fig 10A). Although higher than typical baseline fluctuations, the complex achieved a stable equilibrium between 25 and 31 ns, indicating a transition toward a steady-state conformation [75,76]. The RMSF profiles indicated a higher degree of flexible movement within the complex, evidenced by an average value of 0.640 nm (Fig 10B) [77]. The RG analysis further elucidated the compactness of the system, yielding an average value of 3.957 nm with a stable trajectory observed between 115 and 133 ns (Fig 10C). Interestingly, these findings suggest the vaccine–TLR4 complex exhibits lower compactness compared to its apoprotein form [78]. This is complemented by the SASA results, which averaged 501.11 nm² and remained stable from 75 to 130 ns (Fig 10D). The lower SASA values relative to other configurations suggest that the vaccine–TLR4 complex adopts a more shielded orientation, potentially influencing its molecular engagement and signaling kinetics within the solvent environment.

### Immune simulation analysis

**Fig 11:**
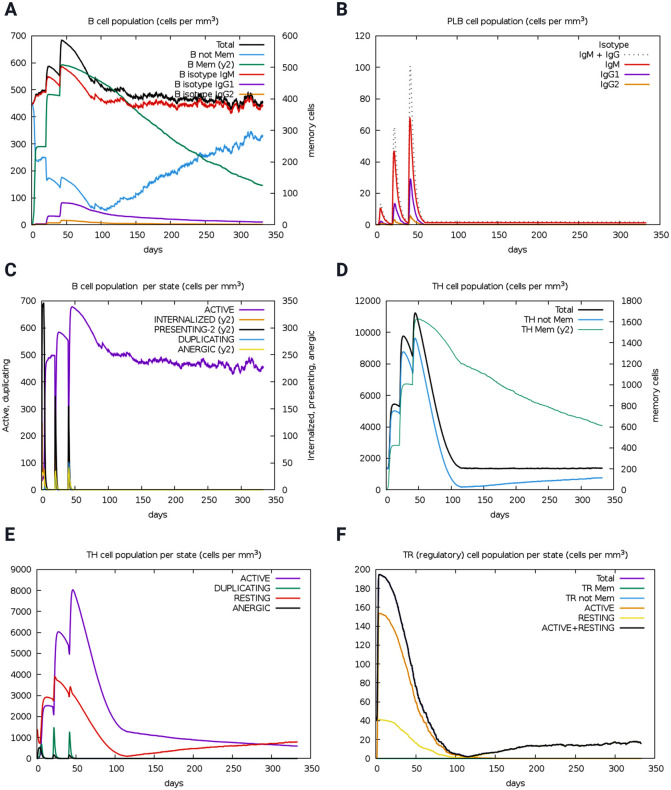
Temporal dynamics of immune cell populations during simulated immune response. (A) Total B-cell population and associated subsets, including non-memory, memory, and antibody isotype-producing B cells (IgG1 and IgG2), over time. (B) Plasma B lymphocyte (PLB) populations producing different antibody isotypes during the immune response. (C) B-cell population dynamics across functional states, including active, internalized, presenting, duplicating, and anergic cells. (D) Total T helper (TH) cell population showing non-memory and memory TH-cell dynamics. (E) TH-cell population distribution across active, duplicating, resting, and anergic states. (F) Regulatory T-cell (TR) population dynamics, including memory, non-memory, active, and resting states, throughout the simulation period. Cell populations are represented as cells per mm³ over 350 days.

**Fig 12:**
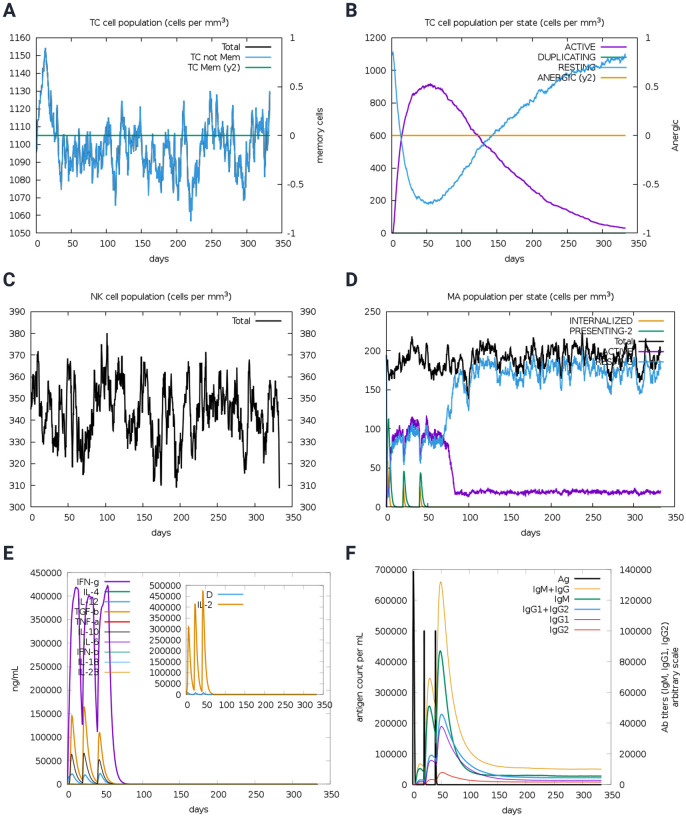
Immune simulation profiling of cellular and humoral immune responses over time. (A) Total cytotoxic T-cell (TC) population, including non-memory and memory TC-cell subsets, during the simulated immune response. (B) TC-cell population dynamics across functional states, including active, duplicating, resting, and anergic cells. (C) Natural killer (NK) cell population fluctuations throughout the simulation period. (D) Macrophage (MA) population dynamics, including active, resting, internalized, and presenting states. (E) Cytokine and interleukin response profiles, showing temporal variations in IFN-γ, IL-4, IL-10, IL-12, IL-18, TNF-β, TGF-β, IFN-β, IL-6, and dendritic cell (D)–associated cytokine responses. (F) Antigen concentration and antibody response kinetics, including IgM, IgG, IgG1, and IgG2 titers, over 350 days. Cell populations are represented as cells per mm³, cytokine levels as ng/mL, and antigen counts per mL.

The graphs of immune simulations depicted in Figs 11, 12, and S3 Fig were retrieved from the C-ImmSim online server. Following three consecutive vaccine doses administered at 20-day intervals, the construct elicited a strong and sustained host immune response. The B-cell population maintained a stable total cell count above 400/mm^3^ after each injection (Fig 11A), while the plasma B lymphocyte (PLB) population also persisted at a relatively constant level over an extended period (Fig 11B). Moreover, the per-state active B-cell population remained consistently within 400–500/mm^3^ after 100 days of vaccination (Fig 11C). The total helper T-cell population stabilized near 1400/mm^3^ approximately four months after the initial dose (Fig 11D). During this period, non-memory helper T-cells gradually increased, whereas memory helper T-cells declined over time. Similarly, the per-state helper T-cell profile demonstrated a gradual reduction in active T-cells after four months compared with the rapid decline observed during the earlier phase, while resting T-cells progressively increased (Fig 11E). In addition, the active and resting regulatory T-cell populations were maintained near 20/mm^3^ approximately six months after the first immunization (Fig 11F), indicating prolonged immune regulation and sustained cellular immune activity.

The immune simulation profiles presented in Fig 12 demonstrated sustained cellular and cytokine-mediated immune responses following repeated vaccine exposure. The total cytotoxic T-cell population remained relatively stable around 1100/mm³ throughout the simulation period (Fig 12A). However, non-memory cytotoxic T-cells exhibited fluctuating trends, whereas memory cytotoxic T-cells remained nearly constant. The per-state cytotoxic T-cell analysis revealed that active cells gradually declined after approximately 100 days, while resting cells steadily increased and became dominant during later stages of the simulation (Fig 12B). The natural killer (NK) cell population fluctuated within a stable range of approximately 320–370/mm³ over the entire simulation period, indicating maintained immune activity (Fig 12C). Likewise, macrophage-associated (MA) populations displayed sustained activation, with the total macrophage count remaining close to 180–210/mm³ after prolonged exposure (Fig 12D). Although active macrophages decreased after nearly 80 days, resting macrophages persisted at elevated levels, suggesting continued immune surveillance.

The cytokine and interleukin profiles further indicated strong immune activation (Fig 12E). Elevated levels of IFN-γ and IL-2 were particularly noticeable following vaccine administration, supporting robust cellular immune responses. Other cytokines, including TNF-β, IL-10, IL-12, and TGF-β, also showed transient increases during the early stages of immune stimulation. Furthermore, antigen concentration rapidly declined following each exposure, concomitant with increased antibody production (Fig 12F). High titers of IgM + IgG, IgG1 + IgG2, and IgM antibodies were maintained over time, indicating efficient antigen clearance and durable humoral immunity.

Moreover, the immune simulation results shown in S3 Fig further confirmed sustained immune activation following vaccination. The dendritic cell (DC) population maintained stable total and resting cell counts within approximately 150–220/mm³ throughout the simulation period, while active DCs remained consistently detectable, indicating continued antigen-processing activity (S3(A) Fig). Likewise, the epithelial cell (EP) population demonstrated stable active cell levels ranging from 350–420/mm³, whereas infected and presenting epithelial cells remained negligible during the observation period (S3(B) Fig). These findings suggest persistent immune surveillance and effective immune regulation after vaccination. These findings validate the immune response-generating efficacy of the designed vaccine candidate.

### Back-translation, codon optimization, and *in silico* cloning

**Fig 13:**
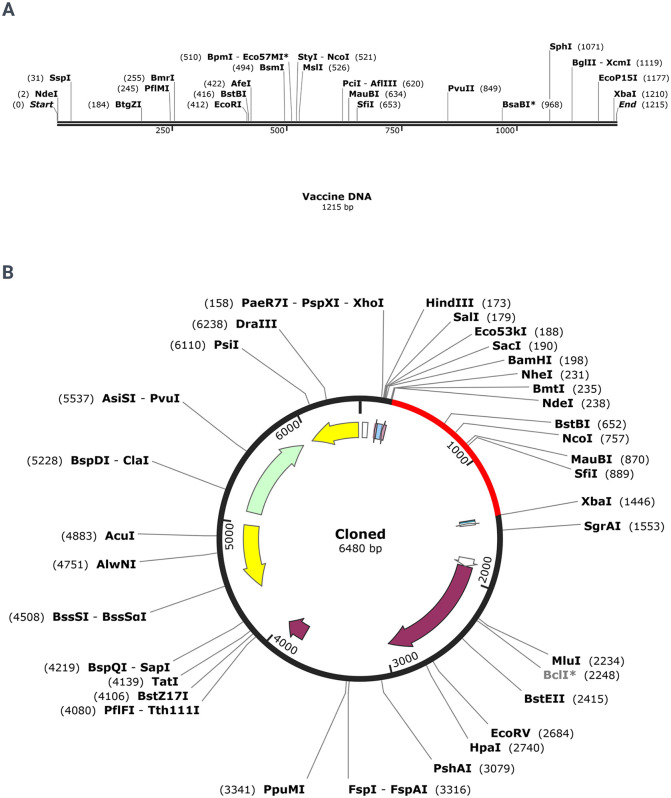
Insertion of the designed vaccine into the pET-28a(+) vector using SnapGene software (www.snapgene.com). (A) The vaccine DNA map after integration of the cut sites. (B) The pET-28a(+) vector map after insertion of the vaccine. The inserted portion iso marked with red color.

EMBOSS Backtranseq provided a back-translated RNA sequence of the vaccine with *Escherichia coli* (strain K12) organism. For *in silico* cloning, *E. coli* expression system plays vital role in expressing vaccines efficiently. The back-translated sequence is composed of 1203 nucleotides against the 401 amino acids of the vaccine (S3 Table). Afterwards, JCAT was employed for the codon usage adaptation. With *E. coli* (strain K12) expression system, the codon adaptation index (CAI) of the improved sequence was observed as 1.0 and the GC content was 53.78%, whereas the optimum range for CAI is 0.8–1.0 and that of GC content is 30–70%. Before improvement, the CAI and GC content were 0.58 and 60.78%, respectively. These measures signify *E. coli* expression system as efficient and good fit for expressing the designed vaccine. Two cut sites for restriction enzymes *Nde*I and *Xba*I were integrated to the terminals of the vaccine and the DNA turned into 1215 bp in length (Fig 13A). Thus, the restriction enzymes *Nde*I and *Xba*I were common in both the vaccine DNA and the vector. Prior to insertion, the positions of these two enzyme sites in the vector were 238 and 335, respectively, whereas the vector was 5369 bp in length. Upon cutting the vaccine DNA and the vector with the same enzymes, the vaccine DNA was successfully inserted into the pET-28a(+) vector using SnapGene software. The final plasmid length turned into 6480 bp (Fig 13B).

## Discussion

Dengue fever can lead to severe flu-like symptoms and even may develop life-threatening dengue hemorrhagic fever or dengue shock syndrome. Every dengue infection carries the risk of hospitalization and severe illness, and if not treated promptly, severe dengue can lead to death [79]. This highlights the importance of vaccination as a preventive measure. The complexity of the DENV, which has four distinct serotypes, poses a significant challenge for vaccine development [80]. Immunity to one serotype does not guarantee protection against the others, and subsequent infections with different serotypes can increase the risk of severe dengue. This necessitates a tetravalent vaccine that can confer immunity against all four serotypes to effectively reduce the global health burden of dengue. Computational approaches have transformed vaccine development by enabling faster, more cost-effective workflows than traditional methods [81]. Immunoinformatics and computational vaccinology analyze viral genomes to identify likely immunogenic components, including B-cell and T-cell epitopes essential for epitope-based vaccine design. These tools also simulate immune responses *in silico*, allowing prediction of vaccine behavior before laboratory or clinical testing, thereby accelerating development and improving response time to emerging infectious diseases.

The designed vaccine construct, comprising 401 amino acids, includes a combination of 23 epitopes—18 T-cell and 5 B-cell epitopes—thus ensuring broad coverage against all four DENV serotypes. The inclusion of human β-defensin 3 as an adjuvant is a novel approach that could enhance the vaccine’s immunogenicity binding to TLR2 and TLR4 which are two significant immune receptors [82]. The vaccine showed good physicochemical properties and solubility which ensures its suitability as a peptide vaccine candidate. The vaccine construct has a molecular weight of 43.84 kDa, and it exhibits a robust stability profile with an instability index of 28.06 which is far below the threshold 40 [43,44]. A GRAVY value of −0.444, suggests a hydrophilic nature. The solubility scores from SOLpro, SoluProt 1.0, and Protein-Sol further corroborate its soluble characteristics, which is essential for effective delivery and distribution within the body [83]. Moreover, antigenicity, allergenicity and toxicity profiles are critical factors for vaccine efficacy and safety. With a score of 0.9319 from VaxiJen 2.0, the vaccine surpasses the threshold for potential antigenicity [30]. The allergenicity assessments from AllerTOP v.2 and AlgPred server classify the vaccine as a probable non-allergen, which is crucial for minimizing adverse reactions. The absence of toxicity, as indicated by the zero results in return from the T3DB server indicates that the vaccine sequence does not match any existing toxins listed in their database [45]. This further supports the vaccine’s safety profile.

Population coverage is a measure of a vaccine’s potential reach and impact. This vaccine has a population coverage of 97.35% worldwide, and thus demonstrates a promising capacity for widespread immunization. Even if considered region-wise, the vaccine shows >90% coverage in major regions of the world (Fig 3). The average hits and PC90 values 2.71 and 1.15, respectively, suggest a broad recognition by the human leukocyte antigen (HLA) system, which is fundamental for eliciting a robust immune response [84].

Disulfide bond is another significant criterion in peptide sequences which contribute to the structure, stability, and function of proteins or peptides [85]. Usually, in a peptide or protein chain, disulfide bonds are formed between two cystine residues by oxidation of the sulfhydryl (-SH) groups [83]. They may be intra-chain and inter-chain (when more than one amino acid chains are present). The presence of disulfide bonds in a vaccine sequence can improve the structural stability as well as the efficacy of a vaccine [86]. The prediction of 4 disulfide bonds within the vaccine sequence suggests a well-defined tertiary structure conducive to proper folding and function of the designed vaccine [87,88]. This structural integrity is vital for the vaccine’s ability to remain structurally stable and induce an appropriate immune response.

The secondary structure prediction for the vaccine peptide, as generated by the PSIPRED 4.0 server, provides a foundational understanding of the protein’s conformational layout [51]. As depicted in S2 Fig, the secondary structure showed high confidence levels in a major portion of its prediction. It predicted the distribution of helices, strands, and coils within the peptide. This distribution is crucial for a protein’s function, hence for the function of our designed vaccine [89]. Afterwards, the tertiary structure modeling and refinement processes through 3Dpro and GalaxyRefine tools, resulted in a polished structure with a favorable Ramachandran plot and GDT-HA scores, indicating a high-quality model suitable for further analysis [90]. The RMSD and MolProbity scores further support the structural integrity of the refined model. Validation of the tertiary structure through PROCHECK with Ramachandran plot analysis revealed a high percentage (93.1%) of residues in the most favored regions, which is indicative of a well-folded protein (Fig 5B) [90]. However, 2 residues were observed to be in the disallowed region. Furthermore, Z-score for the predicted 3D structure were analyzed and a score of −3.83 obtained from the ProSA-web server aligns with the expected range for similar-sized proteins, confirming the model’s validity [90].

The vaccine was subjected to molecular docking analysis afterwards. Molecular docking was performed using ClusPro 2.0 server which automatically detected the most possible binding site of the immune receptors for binding with the adjuvant or vaccine’s core part. The results in molecular docking demonstrated significant interactions between the vaccine model and toll-like receptors TLR2 and TLR4 (Fig 6 and 7). The refined and analytical data from HADDOCK 2.4 and COCOMAPS 2.0 demonstrated significant interactions in between the vaccine and the immune receptors: TLR2 and TLR4 (Fig 8). Adequate van dar Waals force and electrostatic forces were observed in the docked clusters. The area from burried residues were also in expected ranges. The binding energies and the number of members in the docked conformations suggest a stable and potentially effective interaction, which is essential for the vaccine’s ability to elicit an immune response. It is important to note that molecular docking provides structural hypotheses of receptor–ligand interactions and does not demonstrate receptor activation or downstream signaling; therefore, these findings should be interpreted as preliminary and hypothesis-generating.

Afterwards, molecular dynamics (MD) simulation of the vaccine against immune receptors were conducted in GROMACS software. This provide critical insights into the dynamic behavior, stability, flexibility, solvent accessible area, radii of gyration, etc. of the vaccine-receptor complexes, which are essential for understanding their potential efficacy. The RMSD values for the vaccine–TLR2 (average of 1.187 nm) and vaccine–TLR4 (average of 1.913 nm) complexes suggest that the deviations are within acceptable range for both (Fig 9A). However, deviation increase for both the complexes gradually as the simulation progresses and there was observed some major deviation peaks for the vaccine–TLR4 complex which denotes sudden instability. The RMSF analysis indicates that the vaccine–TLR2 complex (average of 0.345 nm) exhibits lower flexibility compared to the vaccine–TLR4 complex (average of 0.436 nm). This flexibility could be advantageous for the complexes, potentially allowing for better accommodation of epitope variations and enhancing immune recognition [91]. The RG values suggest that the vaccine–TLR2 complex (average of 3.99 nm) is slightly more compact than the vaccine–TLR4 complex (average of 3.957 nm). A higher RG value typically correlates with a less compact structure, which may affect the stability and immunogenicity of the complex [92]. Finally, the SASA analysis shows that the vaccine–TLR2 complex has a higher average SASA (510.15 nm^2^) compared to the vaccine–TLR4 complex (501.11 nm^2^). A larger SASA may indicate a higher degree of exposure to solvent molecules, which could facilitate interactions with immune cells and potentially enhance the immunogenic response.

The immune simulation analysis, as depicted in Figs 11, 12 and S3 Fig, provided a quantitative assessment of the host immune response following administration of a novel vaccine candidate. The data, derived from the C-ImmSim online server, underscores the ability of the vaccine to elicit a robust and sustained immune response. The cytokine and interleukin profiles demonstrated strong immune activation by the vaccine sequence. Furthermore, rapid antigen clearance accompanied by sustained IgM and IgG antibody titers indicated effective humoral immune responses and long-term immunological protection. Sustained B-cell populations (400–500 cells/mm³ beyond day 100) and persistently elevated IgM and IgG titers (>10,000 copies/mL) are consistent with the induction of durable humoral immunity — a key correlate of dengue protection, with high-titer neutralizing IgG against the E protein and prM/E junction being the most established serological markers of serotype-specific immunity [92]. The maintenance of helper T-cell levels near 1,400 cells/mm³ reflects the CD4 ⁺ T-cell support required for germinal center reactions, affinity maturation, and long-lived plasma cell differentiation — processes essential for the generation of broadly neutralizing antibody responses against DENV [93]. The cytotoxic T-cell response, peaking around day 13 (1,055–1,155 cells/mm³), aligns with the known role of CD8 ⁺ CTLs in clearing DENV-infected cells and is particularly relevant given that CD8 ⁺ T-cell responses targeting conserved non-structural protein epitopes (especially NS3 and NS4b) have been associated with protection in human cohort studies [94]. The increase of regulatory T-cells to about 20/mm^3^ after 6 months suggests a mechanism to modulate the immune response, preventing potential autoimmunity or hyperactivation. This response is usually expected from a vaccine and it makes the vaccine a good candidate for consideration. Moreover, the fluctuation of cytotoxic T-cells within 1055–1155/mm^3^, peaking around the 13th day, demonstrates their role in the direct elimination of infected cells. The oscillation of NK cells within 309–381/mm^3^ highlights their importance in the early stages of the immune response, providing rapid action against virally infected cells. The consistent levels of IgM and IgG above 10,000/mL throughout the simulation period indicate a strong humoral response, which is critical for neutralizing pathogens and preventing their spread.

Finally, the vaccine was successfully cloned using the pET-28(a)+ vector of *E. coli* (Fig 13). A successful cloning with this vector indicates the vaccines scope in terms of massive production which, in the long run, may make it a cost effective solution if produced industrially. However, before that, the vaccine is to be validated using *in vitro*, *in vivo*, and clinical validations. As the *in silico* results show a positive future prospect through propitious predictions and some pre-validations.

## Conclusion

This study has resulted in the successful development of a tetravalent multi-epitope subunit vaccine for all DENV serotypes. Notably, the vaccine has an antigenicity score of 0.9319 which is very significant. Moreover, with a worldwide population coverage of 97.35%, the vaccine tends to be an efficacious candidate for any ethnicity. The physicochemical properties and solubility of the vaccine were observed in the optimum ranges. The molecular docking results and MD simulations showed propitious interactions, stability, and flexibility with immune receptors (TLR2 and TLR4) of the human body. Furthermore, immune simulations showed that the vaccine is capable of eliciting proper immune responses in the human body for a long time span. Finally, the designed vaccine was successfully cloned by inserting it into the pET-28a(+) vector of *E. coli* (strain K12). Importantly, this vaccine candidate demonstrates promising computational characteristics as a potential vaccine candidate. Since all findings are derived from *in silico* analyses, it requires comprehensive *in vitro* and *in vivo* validations before any conclusions and decisions regarding efficacy or immunogenicity can be established. This study will work as the basis of these future validations that might take it to a new solution against dengue virus.

## Supporting information

S1 Fig Worldwide population coverage graphs retrieved from IEDB Population server. (A) Combined coverage for both MHC-I and MHC-I binding epitopes. (B) MHC-II Binding epitopes’ population coverage. (C) MHC-I Binding epitopes’ population coverage.
(TIF)

S2 Fig Predicted and secondary structure. Helix, strand, and coil regions are shown across the amino acid sequence along with prediction confidence scores.
(TIF)

S3 Fig Immune simulation graphs of the vaccine. (A) Dendritic cells population per state per cubic milimeter of blood. (B) Epithelial cells population per state per cubic milimeter of blood.
(TIF)

S1 Table Complete genome sequences of DENV-1 to −4. The sequences were retrieved from NCBI Genbank.
(DOCX)

S2 Table Population coverage data that were used to build the graphs of S1 Fig. (DOCX)

S3 Table Back-translated and codon-optimized sequence of the vaccine. (DOCX)
