# Supplementary figures and images for: Design of a novel epitope-based tetravalent subunit vaccine against dengue virus: An immunoinformatic approach
Source: PLoS One. 2026 Jul 28;21(7):e0354891. doi: 10.1371/journal.pone.0354891 (PMC13412049; doi:10.1371/journal.pone.0354891)

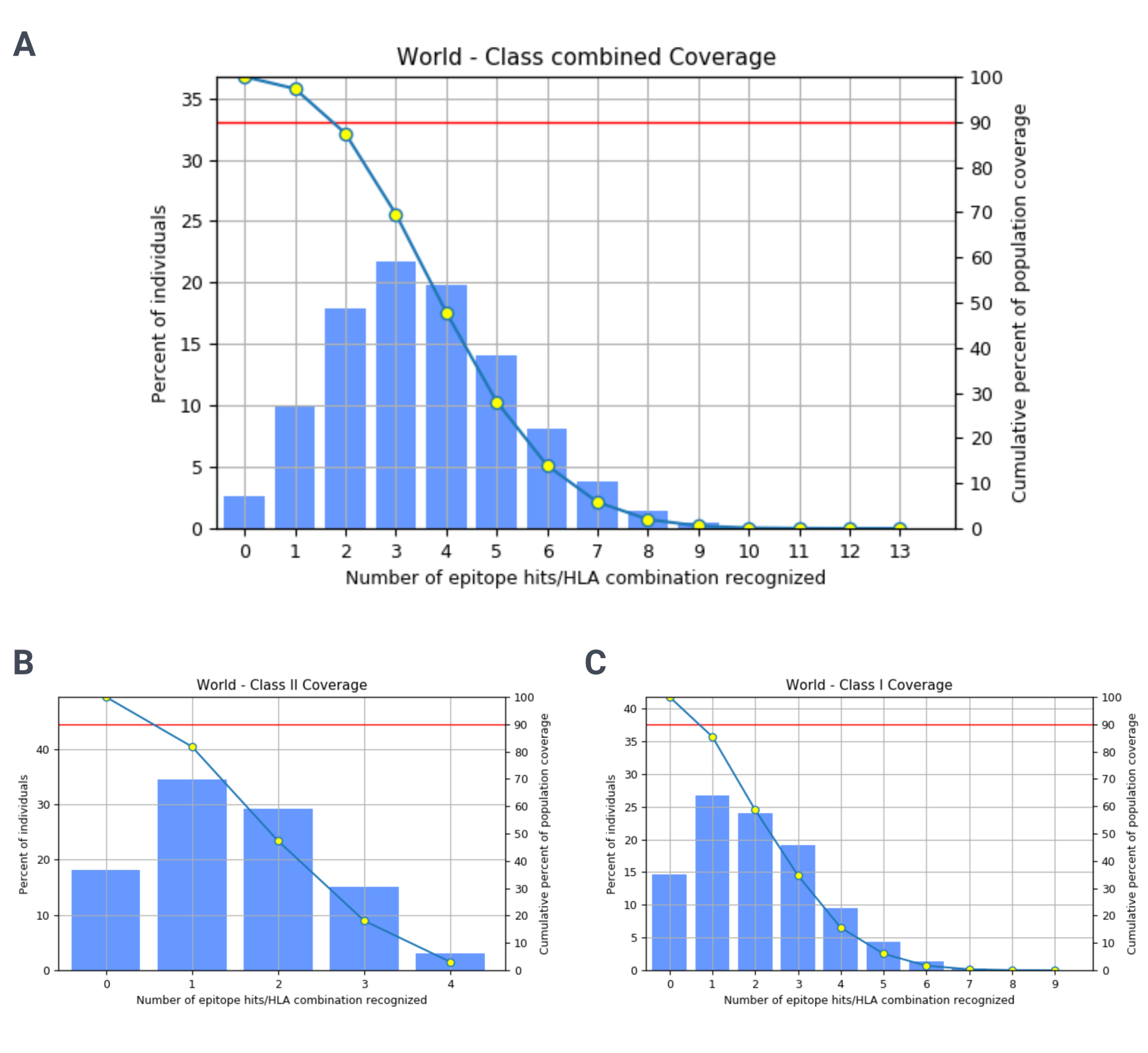

Supplement: S1 Fig — (A) Combined coverage for both MHC-I and MHC-I binding epitopes. (B) MHC-II Binding epitopes’ population coverage. (C) MHC-I Binding epitopes’ population coverage. (TIF) [file pone.0354891.s001.tif]

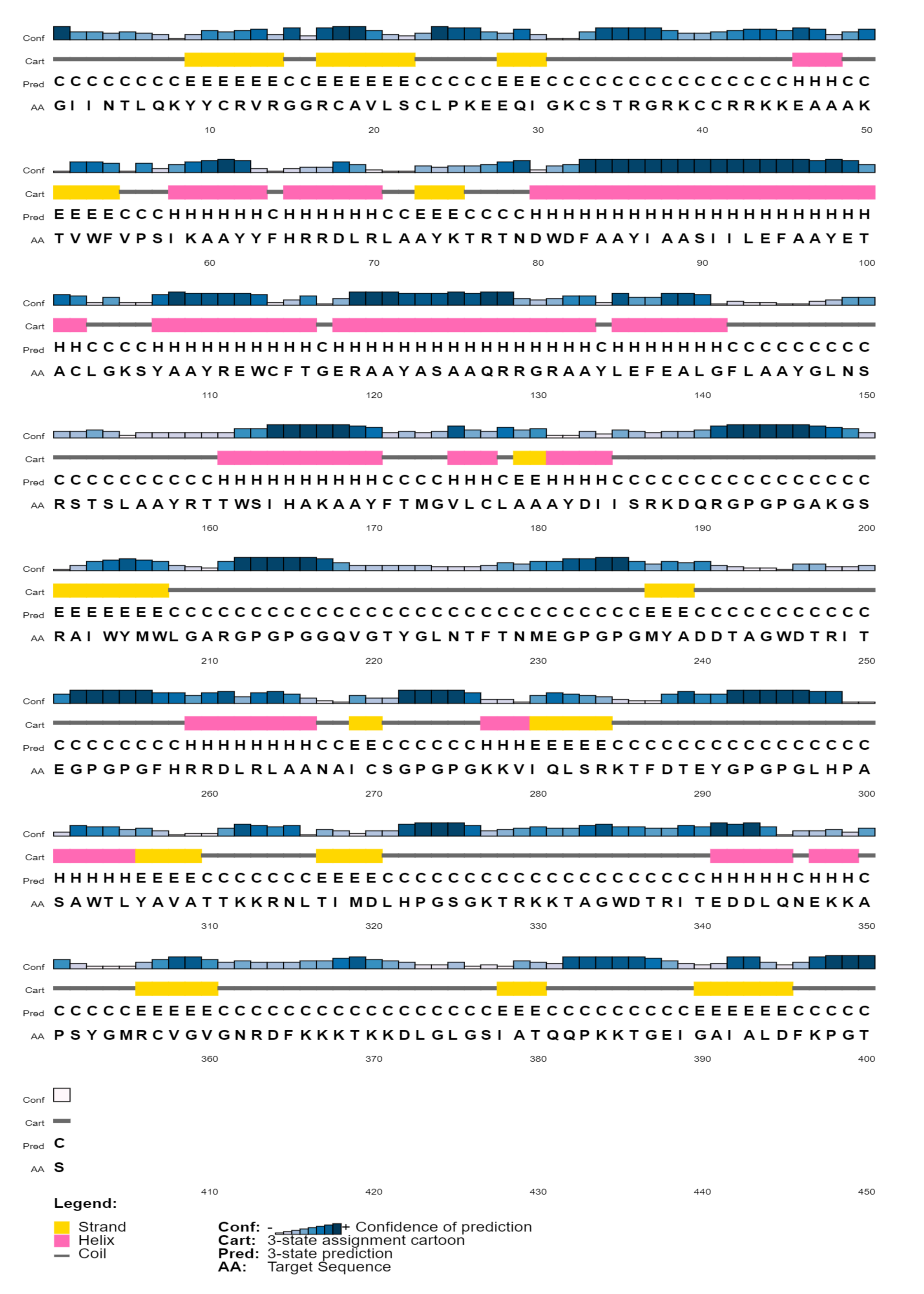

Supplement: S2 Fig — Helix, strand, and coil regions are shown across the amino acid sequence along with prediction confidence scores. (TIF) [file pone.0354891.s002.tif]

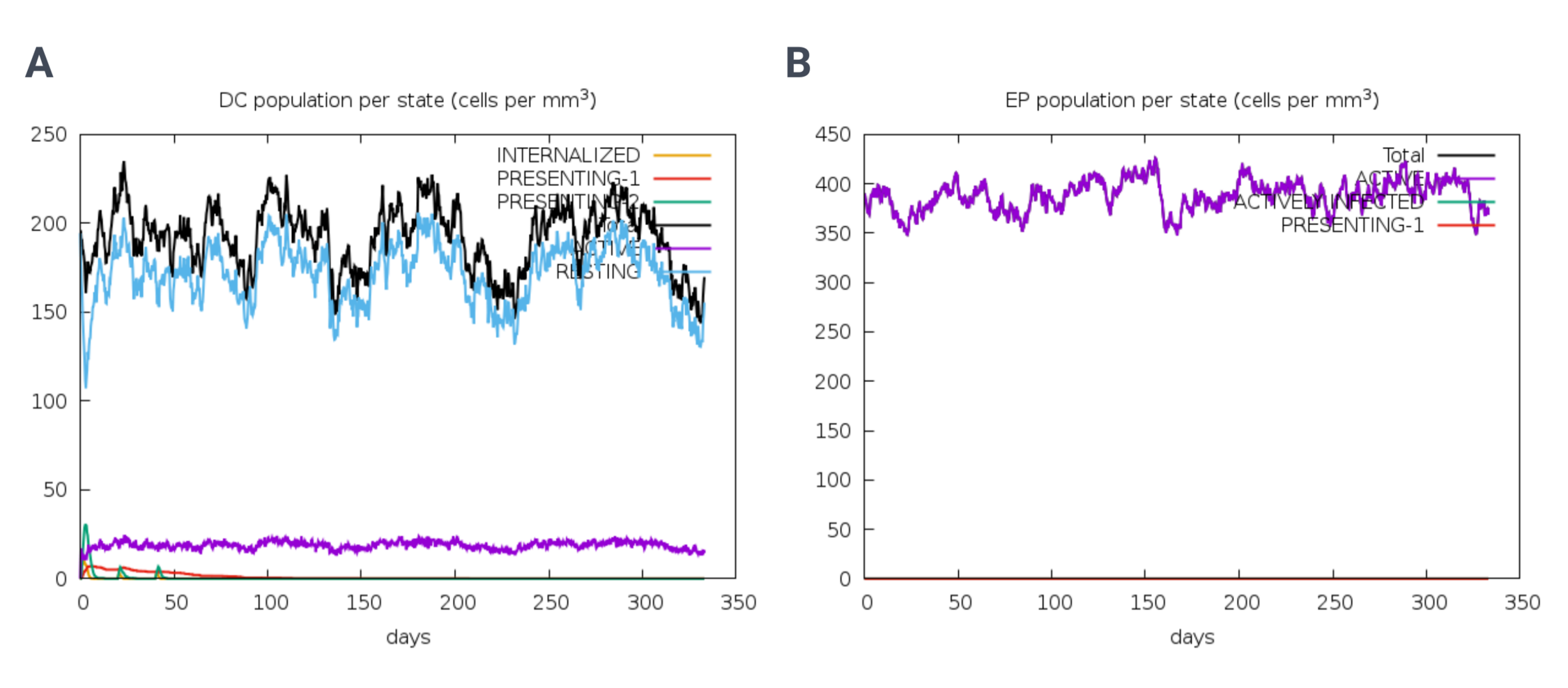

Supplement: S3 Fig — (A) Dendritic cells population per state per cubic milimeter of blood. (B) Epithelial cells population per state per cubic milimeter of blood. (TIF) [file pone.0354891.s003.tif]
