# Supplementary material for: Design of a novel epitope-based tetravalent subunit vaccine against dengue virus: An immunoinformatic approach
Source: PLoS One. 2026 Jul 28;21(7):e0354891. doi: 10.1371/journal.pone.0354891 (PMC13412049; doi:10.1371/journal.pone.0354891)
Supplement: S1 Table — The sequences were retrieved from NCBI Genbank. (DOCX) [file pone.0354891.s004.docx]

**S1 Table. Complete genome sequences of DENV-1 to -4.** The sequences were retrieved from NCBI Genbank.

| **DENV Serotype** | **Amino Acid Sequence Against the Nucleotides** |
| --- | --- |
| DENV1 | MNNQRKKTGRPSFNMLKRARNRVSTVSQLAKRFSKGLLSGQGPM  KLVMAFIAFLRFLAIPPTAGILARWGSFKKNGAIKVLRGFKKEISNMLNIMNRRKRSV  TMLLMLLPTALAFHLTTRGGEPHMIVSKQERGKSLLFKTSAGVNMCTLIAMDLGELCE  DTMTYKCPRITETEPDDVDCWCNATETWVTYGTCSQTGEHRRDKRSVALAPHVGLGLE  TRTETWMSSEGAWKQIQKVETWALRHPGFTVIALFLAHAIGTSITQKGIIFILLMLVT  PSMAMRCVGIGNRDFVEGLSGATWVDVVLEHGSCVTTMAKDKPTLDIELLKTEVTNPA  VLRKLCIEAKISNTTTDSRCPTQGEATLVEEQDTNFVCRRTFVDRGWGNGCGLFGKGS  LITCAKFKCVTKLEGKIVQYENLKYSVIVTVHTGDQHQVGNETTEHGTTATITPQAPT  SEIQLTDYGALTLDCSPRTGLDFNEMVLLTMKKKSWLVHKQWFLDLPLPWTSGASTSQ  ETWNRQDLLVTFKTAHAKKQEVVVLGSQEGAMHTALTGATEIQTSGTTTIFAGHLKCR  LKMDKLILKGMSYVMCTGSFKLEKEVAETQHGTVLVQVKYEGTDAPCKIPFSSQDEKG  VTQNGRLITANPIVTDKEKPVNIEAEPPFGESYIVVGAGEKALKLSWFKKGSSIGKMF  EATARGARRMAILGDTAWDFGSIGGVFTSVGKLIHQIFGTAYGVLFSGVSWTMKIGIG  ILLTWLGLNSRSTSLSMTCIAVGMVTLYLGVMVQADSGCVINWKGRELKCGSGIFVTN  EVHTWTEQYKFQADSPKRLSAAIGKAWEEGVCGIRSATRLENIMWKQISNELNHILLE  NDMKFTVVVGDVSGILAQGKKMIRPQPMEHKYSWKSWGKAKIIGADVQNTTFIIDGPN  TPECPDNQRAWNIWEVEDYGFGIFTTNIWLKLRDSYTQVCDHRLMSAAIKDSKAVHAD  MGYWIESEKNETWKLARASFIEVKTCIWPKSHTLWSNGVLESEMIIPKIYGGPISQHN  YRPGYFTQTAGPWHLGKLELDFDLCEGTTVVVDEHCGNRGPSLRTTTVTGKTIHEWCC  RSCTLPPLRFKGEDGCWYGMEIRPVKEKEENLVKSMVSAGSGEVDSFSLGLLCISIMI  EEVMRSRWSRKMLMTGTLAVFLLLTMGQLTWNDLIRLCIMVGANASDKMGMGTTYLAL  MATFRMRPMFAVGLLFRRLTSREVLLLTVGLSLVASVELPNSLEELGDGLAMGIMMLK  LLTDFQSHQLWATLLSLTFVKTTFSLHYAWKTMAMILSIVSLFPLCLSTTSQKTTWLP  VLLGSLGCKPLTMFLITENKIWGRKSWPLNEGIMAVGIVSILLSSLLKNDVPLAGPLI  AGGMLIACYVISGSSADLSLEKAAEVSWEEEAEHSGASHNILVEVQDDGTMKIKDEER  DDTLTILLKATLLAISGVYPMSIPATLFVWYFWQKKKQRSGVLWDTPSPPEVERAVLD  DGIYRILQRGLLGRSQVGVGVFQEGVFHTMWHVTRGAVLMYQGKRLEPSWASVKKDLI  SYGGGWRFQGSWNAGEEVQVIAVEPGKNPKNVQTAPGTFKTPEGEVGAIALDFKPGTS  GSPIVNREGKIVGLYGNGVVTTSGTYVSAIAQAKASQEGPLPEIEDEVFRKRNLTIMD  LHPGSGKTRRYLPAIVREAIKRKLRTLVLAPTRVVASEMAEALKGMPIRYQTTAVKSE  HTGKEIVDLMCHATFTMRLLSPVRVPNYNMIIMDEAHFTDPASIAARGYISTRVGMGE  AAAIFMTATPPGSVEAFPQSNAVIQDEERDIPERSWNSGYDWITDFPGKTVWFVPSIK  SGNDIANCLRKNGKRVVQLSRKTFDTEYQKTKNNDWDYVVTTDISEMGANFRADRVID  PRRCLKPVILKDGPERVILAGPMPVTVASAAQRRGRIGRNQNKEGDQYIYMGQPLNND  EDHAHWTEAKMLLDNINTPEGIIPALFEPEREKSAAIDGEYRLRGEARKTFVELMRRG  DLPVWLSYKVASEGFQYSDRRWCFDGERNNQVLEENMDVEIWTKEGERKKLRPRWLDA  RTYSDPLALREFKEFAAGRRSVSGDLILEIGKLPQHLTQRAQNALDNLVMLHNSEQGG  KAYRHAMEELPDTIETLMLLALIAVLTGGVTLFFLSGRGLGKTSIGLLCVIASSALLW  MASVEPHWIAASIILEFFLMVLLIPEPDRQRTPQDNQLAYVVIGLLFMILTVAANEMG  LLETTKKDLGIGHAAAENHHHAAMLDVDLHPASAWTLYAVATTIITPMMRHTIENTTA  NISLTAIANQAAILMGLDKGWPISKMDIGVPLLALGCYSQVNPLTLTAAVLMLVAHYA  IIGPGLQAKATREAQKRTAAGIMKNPTVDGIVAIDLDPVVYDAKFEKQLGQIMLLILC  TSQILLMRTTWALCESITLATGPLTTLWEGSPGKFWNTTIAVSMANIFRGSYLAGAGL  AFSLMKSLGGGRRGTGAQGETLGEKWKRQLNQLSKSEFNTYKRSGIIEVDRSEAKEGL  KRGETTKHAVSRGTAKLRWFVERNLVKPEGKVIDLGCGRGGWSYYCAGLKKVTEVKGY  TKGGPGHEEPIPMATYGWNLVKLYSGKDVFFTPPEKCDTLLCDIGESSPNPTIEEGRT  LRVLKMVEPWLRGNQFCIKILNPYMPSVVETLEQMQRKHGGMLVRNPLSRNSTHEMYW  VSCGTGNIVSAVNMTSRMLLNRFTMAHRKPTYERDVDLGAGTRHVAVEPEVANLDIIG  QRIENIKNEHKSTWHYDEDNPYKTWAYHGSYEVKPSGSASSMVNGVVRLLTKPWDVIP  MVTQIAMTDTTPFGQQRVFKEKVDTRTPKAKRGTAQIMEVTARWLWGFLSRNKKPRIC  TREEFTRKVRSNAAIGAVFVDENQWNSAKEAVEDERFWDLVHRERELHKQGKCATCVY  NMMGKREKKLGEFGKAKGSRAIWYMWLGARFLEFEALGFMNEDHWFSRENSLSGVEGE  GLHKLGYILRDISKIPGGNMYADDTAGWDTRITEDDLQNEAKITDIMEPEHALLATSI  FKLTYQNKVVRVQRPAKNGTVMDVISRRDQRGSGQVGTYGLNTFTNMEAQLIRQMESE  GIFSPSELETPNLAERVLDWLKKHGTERLKRMAISGDDCVVKPIDDRFATALTALNDM  GKVRKDIPQWEPSKGWNDWQQVPFCSHHFHQLIMKDGREIVVPCRNQDELVGRARVSQ  GAGWSLRETACLGKSYAQMWQLMYFHRRDLRLAANAICSAVPVDWVPTSRTTWSIHAH  HQWMTTEDMLSVWNRVWIEENPWMEDKTHVSSWEDVPYLGKREDQWCGSLIGLTARAT  WATNIQVAINQVRRLIGNENYLDFMTSMKRFKNESDPEGALW |
| DENV2 | MNNQRKKAKNTPFNMLKRERNRVSTVQQLTKRFSLGMLQGRGPL  KLFMALVAFLRFLTIPPTAGILKRWGTIKKSKAINVLRGFRKEIGRMLNILNRRRRSA  GMIIMLIPTVMAFHLTTRNGEPHMIVSRQEKGKSLLFKTEDGVNMCTLMAMDLGELCE  DTITYKCPLLRQNEPEDIDCWCNSTSTWVTYGTCTTMGEHRREKRSVALVPHVGMGLE  TRTETWMSSEGAWKHVQRIETWILRHPGFTMMAAILAYTIGTTHFQRALIFILLTAVT  PSMTMRCIGMSNRDFVEGVSGGSWVDIVLEHGSCVTTMAKNKPTLDFELIKTEAKQPA  TLRKYCIEAKLTNTTTESRCPTQGEPSLNEEQDKRFVCKHSMVDRGWGNGCGLFGKGG  IVTCAMFRCKKNMEGKVVQPENLEYTIVITPHSGEEHAVGNDTGKHGKEIKITPQSSI  TEAELTGYGTVTMECSPRTGLDFNEMVLLQMENKAWLVHRQWFLDLPLPWLPGADTQG  SNWIQKETLVTFKNPHAKKQDVVVLGSQEGAMHTALTGATEIQMSSGNLLFTGHLKCR  LRMDKLQLKGMSYSMCTGKFKVVKEIAETQHGTIVIRVQYEGDGSPCKIPFEIMDLEK  RHVLGRLITVNPIVTEKDSPVNIEAEPPFGDSYIIIGVEPGQLKLNWFKKGSSIGQMF  ETTMRGAKRMAILGDTAWDFGSLGGVFTSIGKALHQVFGAIYGAAFSGVSWTMKILIG  VIITWIGMNSRSTSLSVTLVLVGIVTLYLGVMVQADSGCVVSWKNKELKCGSGIFITD  NVHTWTEQYKFQPESPSKLASAIQKAHEEGICGIRSVTRLENLMWKQITPELNHILSE  NEVKLTIMTGDIKGIMQAGKRSLRPQPTELKYSWKTWGKAKMLSTESHNQTFLIDGPE  TAECPNTNRAWNSLEVEDYGFGVFTTNIWLKLKEKQDVFCDSKLMSAAIKDNRAVHAD  MGYWIESALNDTWKIEKASFIEVKNCHWPKSHTLWSNGVLESEMIIPKNLAGPVSQHN  YRPGYHTQITGPWHLGKLEMDFDFCDGTTVVVTEDCGNRGPSLRTTTASGKLITEWCC  RSCTLPPLRYRGEDGCWYGMEIRPLKEKEENLVNSLVTAGHGQVDNFSLGVLGMALFL  EEMLRTRVGTKHAILLVAVSFVTLITGNMSFRDLGRVMVMVGATMTDDIGMGVTYLAL  LAAFKVRPTFAAGLLLRKLTSKELMMTTIGIVLLSQSTIPETILELTDALALGMMVLK  MVRNMEKYQLAVTIMAILCVPNAVILQNAWKVSCTILAVVSVSPLLLTSSQQKTDWIP  LALTIKGLNPTAIFLTTLSRTSKKRSWPLNEAIMAVGMVSILASSLLKNDIPMTGPLV  AGGLLTVCYVLTGRSADLELERAADVKWEDQAEISGSSPILSITISEDGSMSIKNEEE  EQTLTILIRTGLLVISGLFPVSIPITAAAWYLWEVKKQRAGVLWDVPSPPPMGKAELE  DGAYRIKQKGILGYSQIGAGVYKEGTFHTMWHVTRGAVLMHKGKRIEPSWADVKKDLI  SYGGGWKLEGEWKEGEEVQVLALEPGKNPRAVQTKPGLFKTNAGTIGAVSLDFSPGTS  GSPIIDKKGKVVGLYGNGVVTRSGAYVSAIAQTEKSIEDNPEIEDDIFRKRRLTIMDL  HPGAGKTKRYLPAIVREAIKRGLRTLILAPTRVVAAEMEEALRGLPIRYQTPAIRAEH  TGREIVDLMCHATFTMRLLSPVRVPNYNLIIMDEAHFTDPASIAARGYISTRVEMGEA  AGIFMTATPPGSRDPFPQSNAPIIDEEREIPERSWNSGHEWVTDFKGKTVWFVPSIKA  GNDIAACLRKNGKKVIQLSRKTFDSEYVKTRTNDWDFVVTTDISEMGANFKAERVIDP  RRCMKPVILTDGEERVILAGPMPVTHSSAAQRRGRIGRNPKNENDQYIYMGEPLENDE  DCAHWKEAKMLLDNINTPEGIIPSMFEPEREKVDAIDGEYRLRGEARKTFVDLMRRGD  LPVWLAYRVAAEGINYADRRWCFDGVKNNQILEENVEVEIWTKEGERKKLKPRWLDAR  IYSDPLALKEFKEFAAGRKSLTLNLITEMGRLPTFMTQKARDALDNLAVLHTAEAGGR  AYNHALSELPETLETLLLLTLLATVTGGIFLFLMSGRGIGKMTLGMCCIITASILLWY  AQIQPHWIAASIILEFFLIVLLIPEPEKQRTPQDNQLTYVVIAILTVVAATMANEMGF  LEKTKKDLGLGSIATQQPESNILDIDLRPASAWTLYAVATTFVTPMLRHSIENSSVNV  SLTAIANQATVLMGLGKGWPLSKMDIGVPLLAIGCYSQVNPITLTAALFLLVAHYAII  GPGLQAKATREAQKRAAAGIMKNPTVDGITVIDLDPIPYDPKFEKQLGQVMLLVLCVT  QVLMMRTTWALCEALTLATGPISTLWEGNPGRFWNTTIAVSMANIFRGSYLAGAGLLF  SIMKNTTNTRRGTGNIGETLGEKWKSRLNALGKSEFQIYKKSGIQEVDRTLAKEGIKR  GETDHHAVSRGSAKLRWFVERNMVTPEGKVVDLGCGRGGWSYYCGGLKNVREVKGLTK  GGPGHEEPIPMSTYGWNLVRLQSGVDVFFIPPEKCDTLLCDIGESSPNPTVEAGRTLR  VLNLVENWLNNNTQFCIKVLNPYMPSVIEKMEALQRKYGGALVRNPLSRNSTHEMYWV  SNASGNIVSSVNMISRMLINRFTMRYKKATYEPDVDLGSGTRNIGIESEIPNLDIIGK  RIEKIKQEHETSWHYDQDHPYKTWAYHGSYETKQTGSASSMVNGVVRLLTKPWDVVPM  VTQMAMTDTTPFGQQRVFKEKVDTRTQEPKEGTKKLMKITAEWLWKELGKKKTPRMCT  REEFTRKVRSNAALGAIFTDENKWKSAREAVEDSRFWELVDKERNLHLEGKCETCVYN  MMGKREKKLGEFGKAKGSRAIWYMWLGARFLEFEALGFLNEDHWFSRENSLSGVEGEG  LHKLGYILRDVSKKEGGAMYADDTAGWDTRITLEDLKNEEMVTNHMEGEHKKLAEAIF  KLTYQNKVVRVQRPTPRGTVMDIISRRDQRGSGQVGTYGLNTFTNMEAQLIRQMEGEG  VFKSIQHLTITEEIAVQNWLARVGRERLSRMAISGDDCVVKPLDDRFASALTALNDMG  KIRKDIQQWEPSRGWNDWTQVPFCSHHFHELIMKDGRVLVVPCRNQDELIGRARISQG  AGWSLRETACLGKSYAQMWSLMYFHRRDLRLAANAICSAVPSHWVPTSRTTWSIHAKH  EWMTTEDMLTVWNRVWIQENPWMEDKTPVESWEEIPYLGKREDQWCGSLIGLTSRATW  AKNIQAAINQVRSLIGNEEYTDYMPSMKRFRREEEEAGVLW |
| DENV3 | MNNQRKKTGKPSINMLKRVRNRVSTGSQLAKRFSKGLLNGQGPM  KLVMAFIAFLRFLAIPPTAGVLARWGTFKKSGAIKVLKGFKKEISNMLSIINQRKKTS  LCLMMILPAALAFHLTSRDGEPRMIVGKNERGKSLLFKTASGINMCTLIAMDLGEMCD  DTVTYKCPHITEVEPEDIDCWCNLTSTWVTYGTCNQAGEHRRDKRSVALAPHVGMGLD  TRTQTWMSAEGAWRQVEKVETWALRHPGFTILALFLAHYIGTSLTQKVVIFILLMLVT  PSMTMRCVGVGNRDFVEGLSGATWVDVVLEHGGCVTTMAKNKPTLDIELQKTEATQLA  TLRKLCIEGKITNITTDSRCPTQGEAVLPEEQDQNYVCKHTYVDRGWGNGCGLFGKGS  LVTCAKFQCLEPIEGKVVQYENLKYTVIITVHTGDQHQVGNETQGVTAEITPQASTTE  AILPEYGTLGLECSPRTGLDFNEMILLTMKNKAWMVHRQWFFDLPLPWASGATTETPT  WNRKELLVTFKNAHAKKQEVVVLGSQEGAMHTALTGATEIQNSGGTSIFAGHLKCRLK  MDKLELKGMSYAMCTNTFVLKKEVSETQHGTILIKVEYKGEDAPCKIPFSTEDGQGKA  HNGRLITANPVVTKKEEPVNIEAEPPFGESNIVIGIGDNALKINWYKKGSSIGKMFEA  TERGARRMAILGDTAWDFGSVGGVLNSLGKMVHQIFGSAYTALFSGVSWVMKIGIGVL  LTWIGLNSKNTSMSFSCIAIGIITLYLGAVVQADMGCVINWKGKELKCGSGIFVTNEV  HTWTEQYKFQADSPKRLATAIAGAWENGVCGIRSTTRMENLLWKQIANELNYILWENN  IKLTVVVGDTLGVLEQGKRTLTPQPMELKYSWKTWGKAKIVTAETQNSSFIIDGPNTP  ECPSASRAWNVWEVEDYGFGVFTTNIWLKLREVYTQLCDHRLMSAAVKDERAVHADMG  YWIESQKNGSWKLEKASLIEVKTCTWPKSHTLWTNGVLESDMIIPKSLAGPISQHNYR  PGYHTQTAGPWHLGKLELDFNYCEGTTVVITESCGTRGPSLRTTTVSGKLIHEWCCRS  CTLPPLRYMGEDGCWYGMEIRPISEKEENMVKSLVSAGSGKVDNFTMGVLCLAILFEE  VLRGKFGKKHMIAGVFFTFVLLLSGQITWRDMAHTLIMIGSNASDRMGMGVTYLALIA  TFKIQPFLALGFFLRKLTSRENLLLGVGLAMATTLQLPEDIEQMANGVALGLMALKLI  TQFETYQLWTALVSLTCSNTIFTLTVAWRTATLILAGVSLLPVCQSSSMRKTDWLPMT  VAAMGVPPLPLFIFSLKDTLKRRSWPLNEGVMAVGLVSILASSLLRNDVPMAGPLVAG  GLLIACYVITGTSADLTVEKAPDVTWEEEAEQTGVSHNLMITVDDDGTMRIKDDETEN  ILTVLLKTALLIVSGIFPYSIPATLLVWHTWQKQTQRSGVLWDVPSPPETQKAELEEG  VYRIKQQGIFGKTQVGVGVQKEGVFHTMWHVTRGAVLTHNGKRLEPNWASVKKDLISY  GGGWRLSAQWQKGEEVQVIAVEPGKNPKNFQTTPGTFQTTTGEIGAIALDFKPGTSGS  PIINREGKVVGLYGNGVVTKNGGYVSGIAQTNAEPDGPTPELEEEMFKKRNLTIMDLH  PGSGKTRKYLPAIVREAIKRRLRTLILAPTRVVAAEMEEALKGLPIRYQTTATKSEHT  GREIVDLMCHATFTMRLLSPVRVPNYNLIIMDEAHFTDPASIAARGYISTRVGMGEAA  AIFMTATPPGTADAFPQSNAPIQDEERDIPERSWNSGNEWITDFAGKTVWFVPSIKAG  NDIANCLRKNGKKVIQLSRKTFDTEYQKTKLNDWDFVVTTDISEMGANFKADRVIDPR  RCLKPVILTDGPERVILAGPMPVTAASAAQRRGRVGRNPQKENDQYIFTGQPLNNDED  HAHWTEAKMLLDNINTPEGIIPALFEPEREKSAAIDGEYRLKGESRKTFVELMRRGDL  PVWLAHKVASEGIKYTDRKWCFDGQRNNQILEENMDVEIWTKEGEKKKLRPRWLDART  YSDPLALKEFKDFAAGRKSIALDLVTEIGRVPSHLAHRTRNALDNLVMLHTSEDGGRA  YRHAVEELPETMETLLLLGLMILLTGGAMLFLISGKGIGKTSIGLICVIASSGMLWMA  EVPLQWIASAIVLEFFMMVLLIPEPEKQRTPQDNQLAYVVIGILTLAATIAANEMGLL  ETTKRDLGMSKEPGVVSPTSYLDVDLHPASAWTLYAVATTVITPMLRHTIENSTANVS  LAAIANQAVVLMGLDKGWPISKMDLGVPLLALGCYSQVNPLTLTAAVLLLITHYAIIG  PGLQAKATREAQKRTAAGIMKNPTVDGIMTIDLDSVIFDSKFEKQLGQVMLLVLCAVQ  LLLMRTSWALCEALTLATGPITTLWEGSPGKFWNTTIAVSMANIFRGSYLAGAGLAFS  IMKSVGTGKRGTGSQGETLGEKWKKKLNQLSRKEFDLYKKSGITEVDRTEAKEGLKRG  ETTHHAVSRGSAKLQWFVERNMVVPEGRVIDLGCGRGGWSYYCAGLKKVTEVRGYTKG  GPGHEEPVPMSTYGWNIVKLMSGKDVFYLPPEKCDTLLCDIGESSPSPTVEESRTIRV  LKMVEPWLKNNQFCIKVLNPYMPTVIEHLERLQRKHGGMLVRNPLSRNSTHEMYWISN  GTGNIVSSVNMVSRLLLNRFTMTHRRPTIEKDVDLGAGTRHVNAEPETPNMDVIGERI  KRIKEEHNSTWHYDDENPYKTWAYHGSYEVKATGSASSMINGVVKLLTKPWDVVPMVT  QMAMTDTTPFGQQRVFKEKVDTRTPRPMPGTRKAMEITAEWLWRTLGRNKRPRLCTRE  EFTKKVRTNAAMGAVFTEENQWDSAKAAVEDEEFWKLVDRERELHKLGKCGSCVYNMM  GKREKKLGEFGKAKGSRAIWYMWLGARYLEFEALGFLNEDHWFSRENSYSGVEGEGLH  KLGYILRDISKIPGGAMYADDTAGWDTRITEDDLHNEEKIIQQMDPEHRQLANAIFKL  TYQNKVVKVQRPTPTGTVMDIISRKDQRGSGQLGTYGLNTFTNMEAQLVRQMEGEGVL  TKADLENPHLLEKKITQWLETKGVERLKRMAISGDDCVVKPIDDRFANALLALNDMGK  VRKDIPQWQPSKGWHDWQQVPFCSHHFHELIMKDGRKLVVPCRPQDELIGRARISQGA  GWSLRETACLGKAYAQMWSLMYFHRRDLRLASNAICSAVPVHWVPTSRTTWSIHAHHQ  WMTTEDMLTVWNRVWIEENPWMEDKTPVTTWENVPYLGKREDQWCGSLIGLTSRATWA  QNIPTAIQQVRSLIGNEEFLDYMPSMKRFRKEEESEGAIW |
| DENV4 | MNQRKKVVRPPFNMLKRERNRVSTPQGLVKRFSTGLFSGKGPLR  MVLAFITFLRVLSIPPTAGILKRWGQLKKNKAIKILIGFRKEIGRMLNILNGRKRSTI  TLLCLIPTVMAFSLSTRDGEPLMIVAKHERGRPLLFKTTEGINKCTLIAMDLGEMCED  TVTYKCPLLVNTEPEDIDCWCNLTSTWVMYGTCTQSGERRREKRSVALTPHSGMGLET  RAETWMSSEGAWKHAQRVESWILRNPGFALLAGFMAYMIGQTGIQRTVFFVLMMLVAP  SYGMRCVGVGNRDFVEGVSGGAWVDLVLEHGGCVTTMAQGKPTLDFELTKTTAKEVAL  LRTYCIEASISNITTATRCPTQGEPYLKEEQDQQYICRRDVVDRGWGNGCGLFGKGGV  VTCAKFSCSGKITGNLVQIENLEYTVVVTVHNGDTHAVGNDTSNHGVTAMITPRSPSV  EVKLPDYGELTLDCEPRSGIDFNEMILMKMKKKTWLVHKQWFLDLPLPWTAGADTSEV  HWNYKERMVTFKVPHAKRQDVTVLGSQEGAMHSALAGATEVDSGDGNHMFAGHLKCKV  RMEKLRIKGMSYTMCSGKFSIDKEMAETQHGTTVVKVKYEGAGAPCKVPIEIRDVNKE  KVVGRIISSTPLAENTNSVTNIELEPPFGDSYIVIGVGNSALTLHWFRKGSSIGKMFE  STYRGAKRMAILGETAWDFGSVGGLFTSLGKAVHQVFGSVYTTMFGGVSWMIRILIGF  LVLWIGTNSRNTSMAMTCIAVGGITLFLGFTVQADMGCVASWSGKELKCGSGIFVVDN  VHTWTEQYKFQPESPARLASAILNAHKDGVCGIRSTTRLENVMWKQITNELNYVLWEG  GHDLTVVAGDVKGVLTKGKRALTPPVSDLKYSWKTWGKAKIFTPEARNSTFLIDGPDT  SECPNERRAWNSLEVEDYGFGMFTTNIWMKFREGSSEVCDHRLMSAAIKDQKAVHADM  GYWIESSKNQTWQIEKASLIEVKTCLWPKTHTLWSNGVLESQMLIPKSYAGPFSQHNY  RQGYATQTVGPWHLGKLEIDFGECPGTTVTIQEDCDHRGPSLRTTTASGKLVTQWCCR  SCTMPPLRFLGEDGCWYGMEIRPLSEKEENMVKSQVTAGQGTSETFSMGLLCLTLFVE  ECLRRRVTRKHMILVVVITLCAIILGGLTWMDLLRALIMLGDTMSGRIGGQIHLAIMA  VFKMSPGYVLGVFLRKLTSRETALMVIGMAMTTVLSIPHDLMELIDGISLGLILLKIV  TQFDNTQVGTLALSLTFIRSTMPLVMAWRTIMAVLFVVTLIPLCRTSCLQKQSHWVEI  TALILGAQALPVYLMTLMKGASRRSWPLNEGIMAVGLVSLLGSALLKNDVPLAGPMVA  GGLLLAAYVMSGSSADLSLEKAANVQWDEMADITGSSPIVEVKQDEDGSFSIRDVEET  NMITLLVKLALITVSGLYPLAIPVTMTLWYMWQVKTQRSGALWDVPSPAATKKAALSE  GVYRIMQRGLFGKTQVGVGIHMEGVFHTMWHVTRGSVICHETGRLEPSWADVRNDMIS  YGGGWRLGDKWDKEEDVQVLAIEPGKNPKHVQTKPGLFKTLTGEIGAVTLDFKPGTSG  SPIINRKGKVIGLYGNGVVTKSGDYVSAITQAERIGEPDYEVDEDIFRKKRLTIMDLH  PGAGKTKRILPSIVREALKRRLRTLILAPTRVVAAEMEEALRGLPIRYQTPAVKSEHT  GREIVDLMCHATFTTRLLSSTRVPNYNLIVMDEAHFTDPSSVAARGYISTRVEMGEAA  AIFMTATPPGATDPFPQSNSPIEDIEREIPERSWNTGFDWITDYQGKTVWFVPSIKAG  NDIANCLRKSGKKVIQLSRKTFDTEYPKTKLTDWDFVVTTDISEMGANFRAGRVIDPR  RCLKPVILPDGPERVILAGPIPVTPASAAQRRGRIGRNPAQEDDQYVFSGDPLKNDED  HAHWTEAKMLLDNIYTPEGIIPTLFGPEREKTQAIDGEFRLRGEQRKTFVELMRRGDL  PVWLSYKVASAGISYEDREWCFTGERNNQILEENMEVEIWTREGEKKKLRPRWLDARV  YADPMALKDFKEFASGRKSITLDILTEIASLPTYLSSRAKLALDNIVMLHTTERGGRA  YQHALNELPESLETLMLVALLGAMTAGIFLFFMQGKGIGKLSMGLITIAVASGLLWVA  EIQPQWIAASIILEFFLMVLLIPEPEKQRTPQDNQLIYVILTILTIIGLIAANEMGLI  EKTKTDFGFYQVKTETTILDVDLRPASAWTLYAVATTILTPMLRHTIENTSANLSLAA  IANQAAVLMGLGKGWPLHRMDLGVPLLAMGCYSQVNPTTLTASLVMLLVHYAIIGPGL  QAKATREAQKRTAAGIMKNPTVDGITVIDLEPISYDPKFEKQLGQVMLLVLCAGQLLL  MRTTWAFCEVLTLATGPILTLWEGNPGRFWNTTIAVSTANIFRGSYLAGAGLAFSLIK  NAQTPRRGTGTTGETLGEKWKRQLNSLDRKEFEEYKRSGILEVDRTEAKSALKDGSKI  KHAVSRGSSKIRWIVERGMVKPKGKVVDLGCGRGGWSYYMATLKNVTEVKGYTKGGPG  HEEPIPMATYGWNLVKLHSGVDVFYKPTEQVDTLLCDIGESSSNPTIEEGRTLRVLKM  VEPWLSSKPEFCIKVLNPYMPTVIEELEKLQRKHGGNLVRCPLSRNSTHEMYWVSGAS  GNIVSSVNTTSKMLLNRFTTRHRKPTYEKDVDLGAGTRSVSTETEKPDMTIIGRRLQR  LQEEHKETWHYDQENPYRTWAYHGSYEAPSTGSASSMVNGVVKLLTKPWDVIPMVTQL  AMTDTTPFGQQRVFKEKVDTRTPQPKPGTRMVMTTTANWLWALLGKKKNPRLCTREEF  ISKVRSNAAIGAVFQEEQGWTSASEAVNDSRFWELVDKERALHQEGKCESCVYNMMGK  REKKLGEFGRAKGSRAIWYMWLGARFLEFEALGFLNEDHWFGRENSWSGVEGEGLHRL  GYILEEIDKKDGDLMYADDTAGWDTRITEDDLQNEELITEQMAPHHKILAKAIFKLTY  QNKVVKVLRPTPRGAVMDIISRKDQRGSGQVGTYGLNTFTNMEVQLIRQMEAEGVITQ  DDMQNPKGLKERVEKWLKECGVDRLKRMAISGDDCVVKPLDERFGTSLLFLNDMGKVR  KDIPQWEPSKGWKNWQEVPFCSHHFHKIFMKDGRSLVVPCRNQDELIGRARISQGAGW  SLRETACLGKAYAQMWSLMYFHRRDLRLASMAICSAVPTEWFPTSRTTWSIHAHHQWM  TTEDMLKVWNRVWIEDNPNMTDKTPVHSWEDIPYLGKREDLWCGSLIGLSSRATWAKN  IHTAITQVRNLIGKEEYVDYMPVMKRYSAPSESEGVL |
