# Supplementary material for: Design of a novel epitope-based tetravalent subunit vaccine against dengue virus: An immunoinformatic approach
Source: PLoS One. 2026 Jul 28;21(7):e0354891. doi: 10.1371/journal.pone.0354891 (PMC13412049; doi:10.1371/journal.pone.0354891)
Supplement: S2 Table — (DOCX) [file pone.0354891.s005.docx]

**S2 Table. Population coverage data that were used to build the graphs of S1 Fig.**

| **Area** | **Number of epitope hits / HLA combinations recognized** | **Percent of individuals** | **Cumulative percent of population coverage** |
| --- | --- | --- | --- |
| Combined | 0 | 2.65 | 100.0 |
|  | 1 | 9.89 | 97.35 |
|  | 2 | 17.85 | 87.45 |
|  | 3 | 21.75 | 69.61 |
|  | 4 | 19.8 | 47.85 |
|  | 5 | 14.1 | 28.05 |
|  | 6 | 8.15 | 13.95 |
|  | 7 | 3.81 | 5.81 |
|  | 8 | 1.45 | 2.0 |
|  | 9 | 0.44 | 0.56 |
|  | 10 | 0.1 | 0.12 |
|  | 11 | 0.02 | 0.02 |
|  | 12 | 0.0 | 0.0 |
|  | 13 | 0.0 | 0.0 |
| MHC Class I | 0 | 14.58 | 100.0 |
|  | 1 | 26.77 | 85.42 |
|  | 2 | 23.98 | 58.65 |
|  | 3 | 19.09 | 34.67 |
|  | 4 | 9.55 | 15.58 |
|  | 5 | 4.38 | 6.03 |
|  | 6 | 1.29 | 1.65 |
|  | 7 | 0.31 | 0.36 |
|  | 8 | 0.05 | 0.05 |
|  | 9 | 0.0 | 0.0 |
| MHC Class II | 0 | 18.19 | 100.0 |
|  | 1 | 34.46 | 81.81 |
|  | 2 | 29.21 | 47.35 |
|  | 3 | 15.07 | 18.14 |
|  | 4 | 3.07 | 3.07 |
