# Supplementary material for: Design of a novel epitope-based tetravalent subunit vaccine against dengue virus: An immunoinformatic approach
Source: PLoS One. 2026 Jul 28;21(7):e0354891. doi: 10.1371/journal.pone.0354891 (PMC13412049; doi:10.1371/journal.pone.0354891)
Supplement: S3 Table — (DOCX) [file pone.0354891.s006.docx]

**S3 Table. Back-translated and codon-optimized sequence of the vaccine.**

| Back-translated sequence in DNA form retrieved from EMBOSS Backtranseq | GGCATTATTAACACCCTGCAGAAATATTATTGCCGCGTGCGCGGCGGCCGCTGCGCGGTG  CTGAGCTGCCTGCCGAAAGAAGAACAGATTGGCAAATGCAGCACCCGCGGCCGCAAATGC  TGCCGCCGCAAAAAAGAAGCGGCGGCGAAAACCGTGTGGTTTGTGCCGAGCATTAAAGCG  GCGTATTATTTTCATCGCCGCGATCTGCGCCTGGCGGCGTATAAAACCCGCACCAACGAT  TGGGATTTTGCGGCGTATATTGCGGCGAGCATTATTCTGGAATTTGCGGCGTATGAAACC  GCGTGCCTGGGCAAAAGCTATGCGGCGTATCGCGAATGGTGCTTTACCGGCGAACGCGCG  GCGTATGCGAGCGCGGCGCAGCGCCGCGGCCGCGCGGCGTATCTGGAATTTGAAGCGCTG  GGCTTTCTGGCGGCGTATGGCCTGAACAGCCGCAGCACCAGCCTGGCGGCGTATCGCACC  ACCTGGAGCATTCATGCGAAAGCGGCGTATTTTACCATGGGCGTGCTGTGCCTGGCGGCG  GCGTATGATATTATTAGCCGCAAAGATCAGCGCGGCCCGGGCCCGGGCGCGAAAGGCAGC  CGCGCGATTTGGTATATGTGGCTGGGCGCGCGCGGCCCGGGCCCGGGCGGCCAGGTGGGC  ACCTATGGCCTGAACACCTTTACCAACATGGAAGGCCCGGGCCCGGGCATGTATGCGGAT  GATACCGCGGGCTGGGATACCCGCATTACCGAAGGCCCGGGCCCGGGCTTTCATCGCCGC  GATCTGCGCCTGGCGGCGAACGCGATTTGCAGCGGCCCGGGCCCGGGCAAAAAAGTGATT  CAGCTGAGCCGCAAAACCTTTGATACCGAATATGGCCCGGGCCCGGGCCTGCATCCGGCG  AGCGCGTGGACCCTGTATGCGGTGGCGACCACCAAAAAACGCAACCTGACCATTATGGAT  CTGCATCCGGGCAGCGGCAAAACCCGCAAAAAAACCGCGGGCTGGGATACCCGCATTACC  GAAGATGATCTGCAGAACGAAAAAAAAGCGCCGAGCTATGGCATGCGCTGCGTGGGCGTG  GGCAACCGCGATTTTAAAAAAAAAACCAAAAAAGATCTGGGCCTGGGCAGCATTGCGACC  CAGCAGCCGAAAAAAACCGGCGAAATTGGCGCGATTGCGCTGGATTTTAAACCGGGCACC  AGC |
| --- | --- |
| Codon-optimized and improved DNA sequence retrieved from JCAT | GGTATCATCAACACCCTGCAGAAATACTACTGCCGTGTTCGTGGTGGTCGTTGCGCTGTT  CTGTCTTGCCTGCCGAAAGAAGAACAGATCGGTAAATGCTCTACCCGTGGTCGTAAATGC  TGCCGTCGTAAAAAAGAAGCTGCTGCTAAAACCGTTTGGTTCGTTCCGTCTATCAAAGCT  GCTTACTACTTCCACCGTCGTGACCTGCGTCTGGCTGCTTACAAAACCCGTACCAACGAC  TGGGACTTCGCTGCTTACATCGCTGCTTCTATCATCCTGGAATTCGCTGCTTACGAAACC  GCTTGCCTGGGTAAATCTTACGCTGCTTACCGTGAATGGTGCTTCACCGGTGAACGTGCT  GCTTACGCTTCTGCTGCTCAGCGTCGTGGTCGTGCTGCTTACCTGGAATTCGAAGCTCTG  GGTTTCCTGGCTGCTTACGGTCTGAACTCTCGTTCTACCTCTCTGGCTGCTTACCGTACC  ACCTGGTCTATCCACGCTAAAGCTGCTTACTTCACCATGGGTGTTCTGTGCCTGGCTGCT  GCTTACGACATCATCTCTCGTAAAGACCAGCGTGGTCCGGGTCCGGGTGCTAAAGGTTCT  CGTGCTATCTGGTACATGTGGCTGGGTGCTCGTGGTCCGGGTCCGGGTGGTCAGGTTGGT  ACCTACGGTCTGAACACCTTCACCAACATGGAAGGTCCGGGTCCGGGTATGTACGCTGAC  GACACCGCTGGTTGGGACACCCGTATCACCGAAGGTCCGGGTCCGGGTTTCCACCGTCGT  GACCTGCGTCTGGCTGCTAACGCTATCTGCTCTGGTCCGGGTCCGGGTAAAAAAGTTATC  CAGCTGTCTCGTAAAACCTTCGACACCGAATACGGTCCGGGTCCGGGTCTGCACCCGGCT  TCTGCTTGGACCCTGTACGCTGTTGCTACCACCAAAAAACGTAACCTGACCATCATGGAC  CTGCACCCGGGTTCTGGTAAAACCCGTAAAAAAACCGCTGGTTGGGACACCCGTATCACC  GAAGACGACCTGCAGAACGAAAAAAAAGCTCCGTCTTACGGTATGCGTTGCGTTGGTGTT  GGTAACCGTGACTTCAAAAAAAAAACCAAAAAAGACCTGGGTCTGGGTTCTATCGCTACC  CAGCAGCCGAAAAAAACCGGTGAAATCGGTGCTATCGCTCTGGACTTCAAACCGGGTACC  TCT |
